# Dithienopyrrole Derivatives with Nitronyl Nitroxide Radicals and Their Oxidation to Cationic High‐Spin Molecules

**DOI:** 10.1002/chem.201905734

**Published:** 2020-02-28

**Authors:** Kubandiran Kolanji, Martin Baumgarten

**Affiliations:** ^1^ Max Planck Institute for Polymer Research Ackermannweg 10 55128 Mainz Germany; ^2^ Current address: Institute of Organic Chemistry Julius-Maximilians-Universität Am Hubland 97074 Würzburg Germany

**Keywords:** dithienopyrrole, high spin molecules, nitronyl nitroxide, radical cation, stable radical

## Abstract

Three 1 N‐phenyl nitronyl nitroxide (NN) 4‐substituted dithieno[3,2‐*b*:2′,3′‐*d*]pyrrole (**DTP**) derivatives with *R*1=4‐phenyl‐, 4H‐, and 4‐methylthiothiophenyl‐ (**R^1^**_**2**_ **DTP‐Ph‐NN**, R^1^=H, Ph and MeSTh) were designed, synthesized and characterized. The electrochemical properties were studied by cyclic voltammetry (CV). All the molecules exhibited two main oxidation peaks, first for radical cation and next for dication formation. The cation and dication formation were also confirmed by UV/Vis absorption spectroscopy for **Ph_2_DTP‐Ph‐NN** and **MeSTh_2_DTP‐Ph‐NN** titrated with *tris*(4‐bromophenyl)aminiumhexachloroantimonate (magic blue). In addition, the cation and dication formation were verified by EPR spectroscopy. Finally, the exchange interactions (*J/k*_B_) of NN and radical cation were calculated by DFT studies.

## Graphical abstract

**Dithienopyrrole derivatives** with phenyl and methylthiothiophenyl connected with phenylnitronylnitroxides were oxidized to their cations forming high spin *S*=1 molecules.

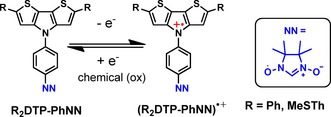

## Introduction

Organic high‐spin molecules are attractive due to their flexible and controllable electronic properties, which can be obtained through different strategies.^1^ High‐spin molecules have been used for various applications such as spintronic devices, and molecular magnets.1d, 2 The spin‐carrying units are very important in the high‐spin molecules, because of the kinetic stability issues of materials for further applications. To enhance the kinetic stability, nitronylnitroxide (NN) and iminonitroxide (IN) were the most recognized spin units for the high‐spin organic molecules.^3^ Organic spin‐carrying units linked with conjugated triarylaminium radical cations are also of interest.^4^ Their combination in mixed stable radical and one electron oxidized cation–diradical systems are more popular, because of their fair kinetic stability and synthetic accessibility. The parent triphenylaminium radical cation is not stable and quickly reacts via the *para* positions to form benzidines. Thus, introduction of an electron donating *para* substituent eliminates this problem. Stable radical units linked with easily oxidizable aromatic amines are of particular interest for triplet ground state high‐spin molecules.4a, 4b, 5 Thus, the mixed stable radical with radical‐cation molecules were provided for high‐spin triplet ground state molecules.1d, 6 A series of nitronyl nitroxide (NN) substituted triarylamine (**TAA‐NN**),^7^ fused arylamine (**FTAA‐NN**),^8^ pyrazine (**NNDPP**),^9^ thianthrene (**TA‐NN**)^10^ phenothiazine (**PTZ‐NN**)^11^ and pyrrole‐based2b molecules and their radical‐cationic forms were reported (Figure S1, Supporting Information (SI)), most of them afforded ground‐state triplet diradicals upon one electron oxidation. In particular, 1‐phenyl NN 4‐substituted‐2,5‐di(thiophen‐2‐yl)‐1*H*‐pyrrole derivatives (**TPT‐Ph‐NN**),^12^ and NN‐substituted conjugated oligomers of dithienyl‐*N*‐methylpyrrole with methoxy substituents at the inner β‐position of thiophene rings type of molecules (**DTP‐P‐NN**),^13^ were utilized for the synthesis of cationic high‐spin molecules. Therefore our interest focused on the synthesis of stable‐radical species substituted with π‐conjugated electron donor systems and their radical cation formation for high‐spin formation. Dithieno[3,2‐*b*:2,3‐*d*]‐pyrrole (**DTP**) derivatives are leading to better π‐conjugation and lower ionization potential through the electron‐donating nature of the molecules. They also have two active positions at the thiophene, which serve to modify the energy levels to substitute different donor and/or acceptor units, which can assist in the control of the HOMO and LUMO energy levels of the molecules. In our molecular design, two‐phenyl (Ph) or ‐methylthiothiophene (MeSTh) groups were introduced to tune the electronic properties of the molecules in the DTP π‐unit.^14^

We report the design, synthesis, and structural characterization of three, NN substituted **DTP** molecules with *R*1=4‐phenyl‐, 4H‐, and 4‐di(methylthiothiophenyl dithieno[3,2‐*b*:2′,3′‐*d*]pyrrole derivatives (**R^1^**_**2**_ **DTP‐Ph‐NN** R^1^=H, Ph and MeSTh) together with the formation of their radical cationic (**R^1^**_**2**_ **DTP‐Ph‐NN)^.+^** forms. Furthermore, intra‐molecular exchange interactions were examined by DFT calculation.

## Results and Discussion

### Molecular design with DFT calculations

**Figure 1:**
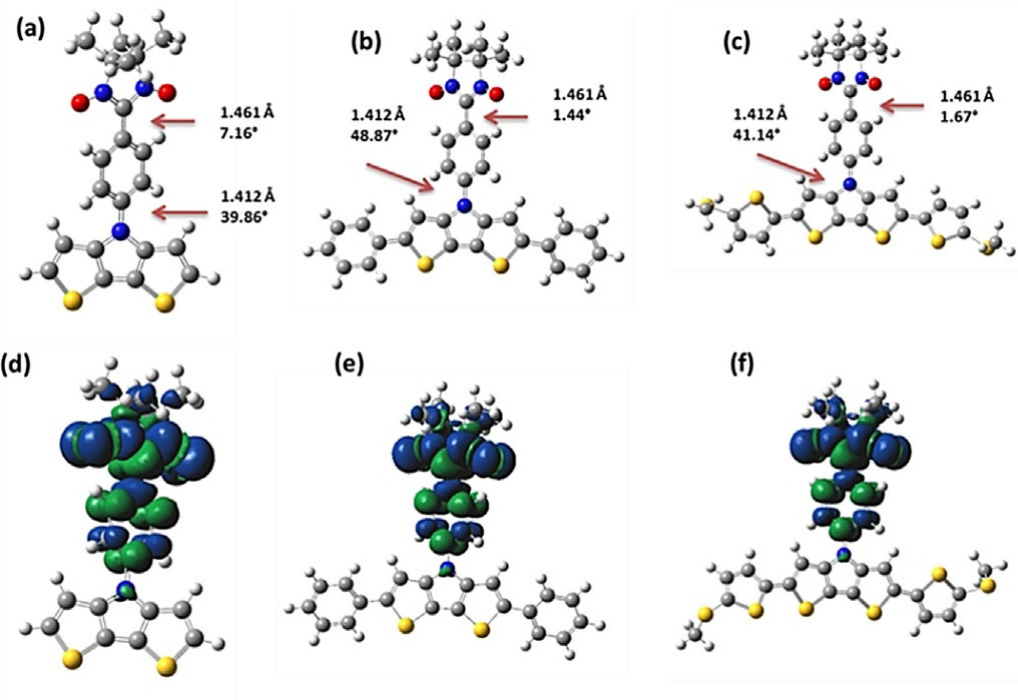
Optimized structures (a), (b), and (c), spin density distributions (d), (e), and (f) for **DTP‐Ph‐NN**, **Ph_2_DTP‐Ph‐NN**, and **MeSTh_2_DTP‐Ph‐NN**, respectively, calculated by DFT using ub3lyp/6–31 g(d) basis set.

The computations were carried out to understand the electronic structure of the molecules. All the DFT calculations were performed using the Gaussian 09 package.^15^ The full geometry optimizations were carried out by UB3LYP/6‐31G(d) level for all the neutral radical molecules. The optimized structures and spin density distribution of the neutral radical molecules of **DTP‐Ph‐NN**, **Ph_2_DTP‐Ph‐NN**, and **MeSTh_2_DTP‐Ph‐NN** are shown in Figure 1.

**Figure 2:**
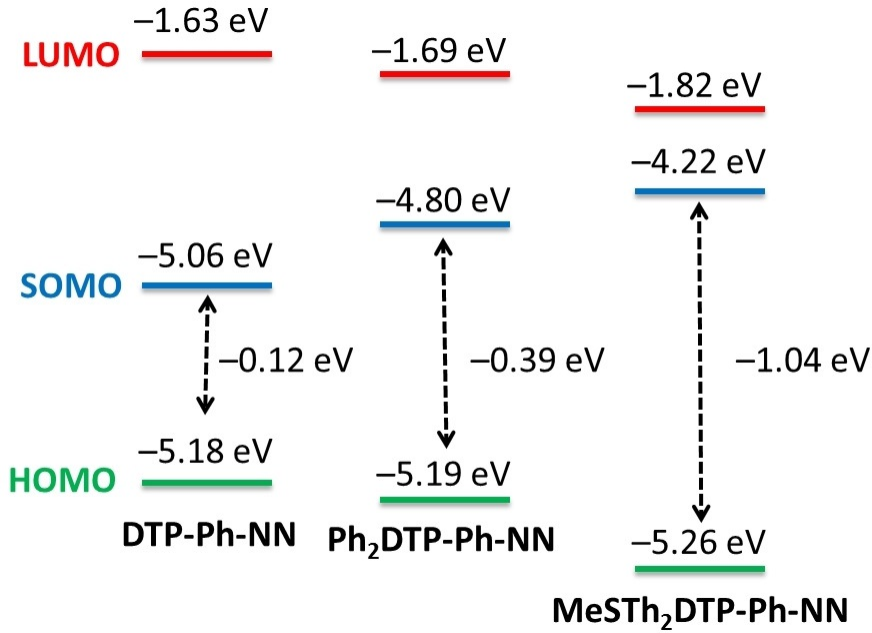
Relative energy levels of the HOMO green line, LUMO blue line, and SOMO red line diagram of the **DTP‐Ph‐NN**, **Ph_2_DTP‐Ph‐NN**, and **MeSTh_2_DTP‐Ph‐NN**, respectively.

In order to get triplet ground state high‐spin molecules, one electron could be oxidized from HOMO of the neutral molecules. The proposed one‐electron oxidation mechanism is described in Figure S14 for spin polarized donor radicals. A similar mechanism was also reported using molecular orbital theory to analyze one‐electron oxidation of the amine‐based spin‐polarized donor radical and pyrrole derivative molecules.3b Therefore, the energy level of the neutral molecules were analyzed using DFT calculations. The energy levels of the HOMO, LUMO, and SOMO were summarized in the Figure 2 and Table S1. The HOMO of the **DTP‐Ph‐NN** (−5.18 eV) is higher energy than HOMO (−5.19 eV) of the **Ph_2_DTP‐PhNN** and **MeSTh_2_DTP‐Ph‐NN** (−5.26 eV). The energy level of the SOMO for **DTP‐Ph‐NN** (−5.06 eV) is lower than for **Ph_2_DTP‐PhNN** SOMO (−4.85 eV) and **MeSTh_2_DTP‐Ph‐NN** (−4.22 eV). The energy difference between HOMO and SOMO are gradually increased for **DTP‐Ph‐NN**, **Ph_2_DTP‐PhNN** and **MeSTh_2_DTP‐Ph‐NN**, respectively. The results clearly indicate that the substitutions on their terminal sites in the core DTP π‐unit with two‐Ph or ‐MeSTh groups were changed the electronic properties of the molecules.

The bond distance between NN and Ph unit is 1.461 Å and Ph and DTP‐backbone is 1.412, these values are same for all the derivatives. The torsion between NN and its attached phenyl is slightly varies for different derivatives such as 7.2°, 1.4°, and 1.7°, similarly between phenyl and DTP‐backbone also varies as 39.9, 48.9, and 41.1 for **DTP‐Ph‐NN**, **Ph_2_DTP‐Ph‐NN**, and **MeSTh_2_DTP‐Ph‐NN**, respectively.

### Synthesis

**Scheme 1:**
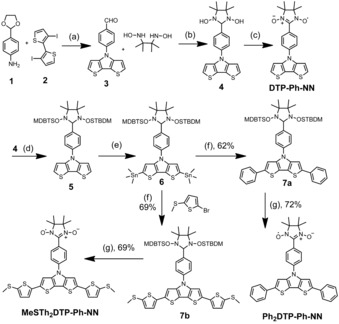
Synthesis of NN substituted **DTP** molecules with *R*1=4‐phenyl‐, 4H‐, and 4‐methylthiothiophenyl dithieno[3,2‐*b*:2′,3′‐*d*]pyrrole derivatives of **R^1^**_**2**_ **DTP‐Ph‐NN** (R=H, Ph and MeSTh). Reaction conditions: (a) Pd_2_(dba)_3_, *t*Bu_3_ONa, *t*Bu_3_P, toluene,125 °C, 46 %, (b) BHA, DMF, 65 C, 52 %, (c) NaIO_4_, DCM, H_2_O, 0 °C, 62 %, (d) *t*BuMe_2_SiCl, imidazole, 55 °C, DMF, 83 %, (e) *n*BuLi, THF, −20 °C, Me_3_SnCl, THF, 91 %, (f) bromobenzene, Pd_2_dba_3_, P(*o*‐tolyl)_3_, toluene, 90 °C, (f) TBAF, THF.

The syntheses of **R_2_DTP‐Ph‐NN**s are demonstrated in Scheme 1. Compound **1** 16 and **2** 17 were prepared by literature procedure and **3** obtained by modified procedure with improved yield.^18^ Ullman condensation of **3** with 2,3‐bis(hydroxylamino)‐2,3‐dimethylbutane (BHA) yielded **4**.^19^ Oxidation of **4** by NaIO_4_ offered **DTP‐Ph‐NN**. Then N−OH groups were protected for **4** with *tert*‐butyldimethylsilyl (TBDMS) through *tert*‐butyldimethylsilyl chloride (*t*BuMe_2_SiCl) in the presence of imidazole in DMF as solvent to afford **5**.^20^ Furthermore, compound **5** was reacted with *n‐*butyl‐lithium solution followed by trimethyltinchloride (Me_3_SnCl) yielding **6**. Stille coupling was carried out between **6** and bromobenzene, or 2‐bromo‐5‐(methylthio)thiophene^21^ yielding **7 a** and **7 b**, respectively. Furthermore, **7 a** or **7 b** were reacted with tetrabutylammoniumfluoride (TBAF) to yield **Ph_2_DTP‐Ph‐NN** and **MeSTh_2_DTP‐Ph‐NN**.^20^ All the diamagnetic precursors were characterized by NMR paramagnetic neutral radical molecules by UV/Vis EPR spectroscopy and HRMS mass spectrometry.

### X‐ray crystallographic studies

The crystal structure analysis is important to understand magnetic interactions in the solid state. Single crystals suitable for X‐ray diffraction analysis were obtained by slow evaporation of CH_2_Cl_2_ solution for **MeSTh_2_DTP‐Ph‐NN** and mixtures of CH_2_Cl_2_ and PhCN solution for **Ph_2_DTP‐Ph‐NN**.

**Figure 3:**
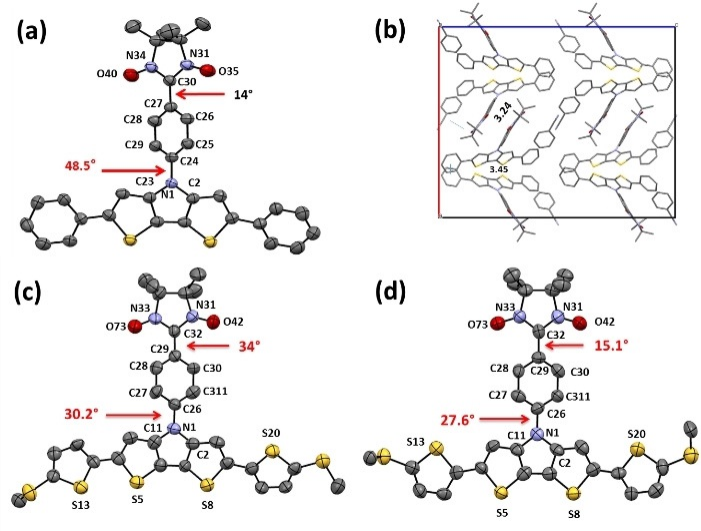
X‐ray crystal structure of (a) **Ph_2_DTP‐Ph‐NN**, (b) molecular packing, (c) and (d) **MeSTh_2_DTP‐Ph‐NN** of the conformers; hydrogen atoms and PhCN for **Ph_2_DTP‐Ph‐NN** are omitted for clarity.

The blue plate‐like **Ph_2_DTP‐PhNN** was crystallized with PhCN solvent molecule in orthorhombic, Pbcn space group. The structure of the molecules is given in the Figure 3 a. The torsions between the radical NN and phenyl are 14.0°. The torsions between the central phenyl and π‐unit is 48.5°. Furthermore, the molecular packing is displayed in Figure 3 b, providing a short intermolecular distance (3.24 Å) between two oxygen atoms; in addition, short π–π intermolecular distances were found to be 3.45 Å for S11⋅⋅⋅C5 and 3.48 Å for S11⋅⋅⋅C4.

The green block **MeSTh_2_DTP‐Ph‐NN** was crystallized in dimeric triclinic form with P−1 space group (Figure S3a). The two structures of the unit cell are given in Figure 3 c and d. The two independent molecules differ mainly in the orientation of the thiophene unit and the torsion angles. In **MeSTh_2_DTP‐PhNN‐A**, the sulfur atoms are arranged as S5 and S13 in *syn*‐orientations, S8 and S20 in anti‐orientations while in molecule **MeSTh_2_DTP‐Ph‐NN‐B**, both S5 and S13, then S8 and S20 are arranged in anti‐orientations. The torsions between the radical NN and Ph are also different in both molecules 34.0° and 15.1° for **MeSTh_2_DTP‐Ph‐NN‐A**, and **MeSTh_2_DTP‐PhNN‐B**, respectively. Similarly slightly different torsion angles were found between the center Ph and DTP core as 30.2° and 27.6° for **MeSTh_2_DTP‐Ph‐NN‐A**, and **MeSTh_2_DTP‐Ph‐NN‐B**, correspondingly. These variations are due to intermolecular interaction present between the molecules in the molecular packing.

### Cyclic voltammetry studies

**Figure 4:**
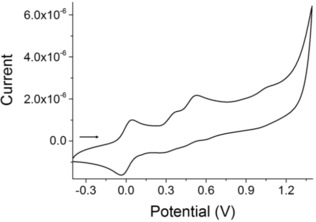
Cyclic voltammograms in PhCN solution of **Ph_2_DTP‐Ph‐NN** at a 0.1 V s^−1^ scan rate, with 0.1 m (*n*‐C_4_H_9_)_4_NBF_4_ as supporting electrolyte, versus ferrocene/ferrocenium (Fc/Fc^+^).

A prerequisite for generating a radical cationic molecule is that the arylamine of DPT moiety has a lower oxidation potential than those of the NN radical. Hence, the electrochemical properties of all the molecules were investigated by the cyclic voltammetry in acetonitrile for **R^1^**_**2**_ **DTP‐Ph‐NN**, (R^1^=H, and Th), and benzonitrile for **Ph_2_DTP‐PhNN** at room temperature. The cyclic voltammograms are given in Figure 4 for **Ph_2_DTP‐Ph‐NN**, and for **R^1^**_**2**_ **DTP‐Ph‐NN**, **(**R^1^=H, and Th in Figure S4). The oxidative process with half‐wave potentials are summarized versus ferrocene/ferrocenium (Fc/Fc^+^) in Table 1.

**Table 1:** Oxidation potentials of half‐wave potentials for **R_2_DTP‐Ph‐NN**.

	*E*_1/2_^ox1^	*E*_1/2_^ox2^	*E*_1/2_^ox3^
**DTP‐Ph‐NN**	+0.38	+0.78	+1.14
**Ph_2_DTP‐Ph‐NN**	+0.33	+0.48	+1.00
**MeSTh_2_DTP‐Ph‐NN**	+0.23	+0.41	+1.11

Oxidative process with half‐wave potentials versus Fc/Fc^+^ in V with (±0.02 V).

**Scheme 2:**
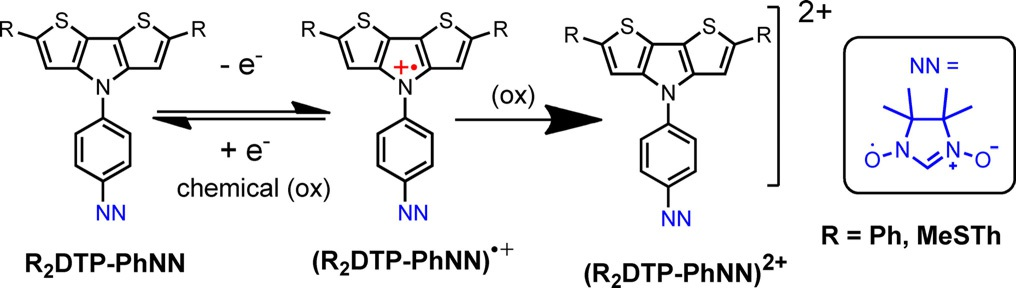
Oxidation and reduction of the **R_2_DTP‐Ph‐NN** and oxidation of (**R_2_DTP‐Ph‐NN)^+.^** molecules.

The first oxidation wave occurs at +0.23, and +0.33, (±0.02) V versus Fc/Fc^+^, for **Th_2_DTP‐Ph‐NN** and **Ph_2_DTP‐Ph‐NN** respectively. The first oxidation potentials were apparently lower than those of the NN unit,2b, 12 and similar compounds of **DTP‐Ph**, **Ph_2_DTP‐Ph**, and **MeSTh_2_DTP‐Ph** reported in the literature.14d–14f All the molecules showed two reversible and one irreversible oxidation waves. The second and third oxidation waves probably correspond to the oxidation process of the NN groups and/or the oxidation process of the **DTP** core which allotted for the dication formation. The **DTP‐Ph‐NN** was polymerized and/or some other side reaction occurred at 0.8 V and 1.1 V (Figure S4b). The extension of the π‐bridge is beneficial for better donor ability to the π‐core. These two molecules exhibited both the first and second oxidation potentials lower than those of the NN radical. It means that the first and second steps were radical cation and dication formations in the oligomer moieties (Scheme 2).

### UV/Vis absorption studies

The optical properties were studied for **R^1^**_**2**_ **DTP‐Ph‐NN** (**R^1^**=**H**, **Ph** and **MeSTh**) by UV/Vis spectroscopy. The absorption spectra are displayed in Figure S5. Two main absorption bands appeared, one around 280–450 nm for π–π* transitions of the donor π‐unit and another about 500–750 nm for *n*–π* transitions of NN radical units which is similar to typically reported nitronylnitroxide molecules.^20^

**Figure 5:**
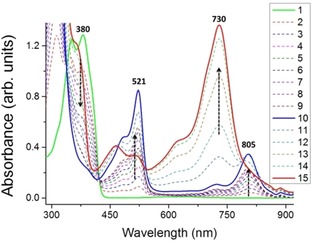
UV/Vis absorption spectra of **Ph_2_DTP‐Ph‐NN** in toluene (≈10^−4^ m) solution (solid green line) and its oxidation by addition of magic blue in CH_2_Cl_2_ at room temperature. Note: broken lines are formation of intermediates; the solid blue line represents monoradical cations; and the solid red line represents the dications.

The chemical oxidation reactions were conducted by *tris*(4‐bromophenyl)aminiumhexachloroantimonate (magic blue, (BrC_6_H_4_)_3_N^.+^SbCl_6_^−^) as the oxidant at room temperature in air, and these reactions were monitored by UV/Vis absorption spectroscopy. During first oxidation two new peaks were formed at 521 and 805 nm for **Ph_2_DTP‐Ph‐NN** (Figure 5) and at 539 and 945 nm for **MeSTh_2_DTP‐Ph‐NN** (Figure S6) due to production of radical cation, at the mean time the absorption band at 380 for **Ph_2_DTP‐Ph‐NN** and 425 for **MeSTh_2_DTP‐Ph‐NN** for the neutral compounds were superseded. The absorption in the longer wavelength regions was assigned as the HOMO‐SOMO and shorter for SOMO‐LUMO transition, respectively. For second oxidation, another new peak appeared at 730 nm for **Ph_2_DTP‐Ph‐NN** and 887 nm for **MeSTh_2_DTP‐Ph‐NN** and former peaks decreased.

### EPR studies

The EPR spectra of neutral radicals of **DTP‐Ph‐NN**, **Ph_2_DTP‐Ph‐NN**, and **MeSTh_2_DTP‐Ph‐NN** in toluene (∼10^−4^ m) in argon‐saturated solutions were measured at room temperature and in frozen solution at 130 K. The experimental and simulated spectra are displayed in Figure S7a–c. All the molecules showed five equally separated lines at room temperature assigned to hyperfine coupling of two equivalent nitrogen nuclei of the NN unit. The *g* values (*g=*2.0070, ±0.02) are nearly the same for all three neutral radicals. The variable temperature EPR spectra of the **Ph_2_DTP‐Ph‐NN** and **Th_2_DTP‐Ph‐NN** are displayed in Figure S9. Moreover, the frozen solution EPR spectra are asymmetric with anisotropic components, providing different numbers of shoulders on the low and high field range (Figure S7d–f). Initially, we tried to oxidize using magic blue as an oxidant in the oxidation process, but the reaction could not be monitored clearly by EPR spectroscopy. It may be that the unreacted magic blue affected the resolution of the spectra. Furthermore, the chemical oxidation reaction was carried out for all the neutral radicals by the silver hexafluoroantimonate (AgSbF_6_) and the reaction was monitored with EPR spectroscopy. **DTP‐Ph‐NN** in CH_2_Cl_2_ (blue solution) was titrated with AgSbF_6_ as an oxidant and the spectra are given in Figure S8a. During addition of the oxidant, the blue solution of **DTP‐Ph‐NN** turned to green and the intensity of the five line spectra decreased. Finally, all the EPR lines disappeared, which is due to decomposition of the NN, and polymerization of the molecules or some other side reaction occurred.

**Figure 6:**
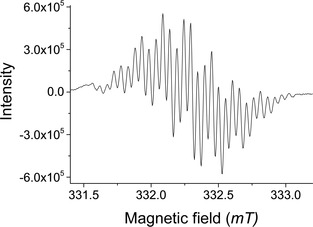
EPR spectra of the (**Ph_2_DTP‐Ph‐NN**)**^.+^** biradical cation monomer obtained by chemical oxidation titrated with AgSbF_6_ as an oxidant in CH_2_Cl_2_ at room temperature.

The blue solution of **Ph_2_DTP‐Ph‐NN** in CH_2_Cl_2_ become brown during addition of the AgSbF_6_, in which the EPR spectrum was recorded, there is a clear change in the spectrum where the center is shifted upfield and the total width narrowed as given in Figure S8b and Figure 6. After addition of one equivalent oxidant, the 30 line EPR spectrum was obtained (Figure 6), once the neutral precursor was consumed. The solution was cooled to 160 K to minimize decomposition of the biradical cations (Figure S10b). The one very weak signal was found at 160 K, this is due to dimerization occurring at lower temperatures. Therefore, no zero field splitting could be observed. After heating, the variable temperature EPR spectrum for the oxidized species of the (**Ph_2_DTP‐Ph‐NN)^.+^** was measured (Figure S10b). While decreasing the temperature, the intensity of the EPR line also decreased until at 200 K, It is just above the melting point (176.5 K) of CH_2_Cl_2_. For **MeSTh_2_DTP‐Ph‐NN** in CH_2_Cl_2_ the light blue/green solution became dark green during addition of the AgSbF_6_, and formed a dark‐green precipitate. The poorly soluble materials might be dimerized radical cations. The new EPR line appeared during addition of AgSbF_6_ along with **MeSTh_2_DTP‐Ph‐NN** (5 line) but the spectra are not resolved, as demonstrated in Figure S8d. The π‐dimerization of dithienylpyrrole radical cation molecules is known from the literature.^13^, ^22^ Further addition of one more equivalent of the AgSbF_6_, kept at −10 °C for 6 h, five‐line EPR spectra were retained for **Ph_2_DTP‐Ph‐NN** and **MeSTh_2_DTP‐Ph‐NN**, the spectra for which are given in Figure S8e,f. These results indicate the dication formation on the DTP core without decomposition of the NN radical.

### Magnetic interaction calculations using DFT calculations

**Figure 7:**
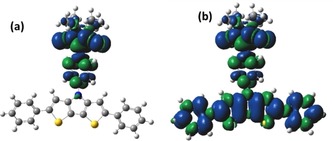
Spin density distributions of the (a) **Ph_2_DTP‐PhNN** and (b) **(Ph_2_DTP‐PhNN)^.+^**.

The intra molecular interaction (*J*_intra,calc_) of NN and radical‐cation was calculated for (**R^1^**_**2**_ **DTP‐Ph‐NN**)**^.+^** for all the molecules with density functional theory (DFT) and hybrid function BLYP, and basis set 6‐31G(d) in the gas phase using the Gaussian 09 package.^15^ The X‐ray structure geometry of **Ph_2_DTP‐Ph‐NN**, **MeSTh_2_DTP‐Ph‐NN** and optimized geometry for **DTP‐Ph‐NN** with positive charge were used for the calculations. The *J*_intra,calc_ values are −3580 K, +5000 K, +965 K for **DTP‐Ph‐NN**, **Ph_2_DTP‐Ph‐NN**, and **MeSTh_2_DTP‐Ph‐NN**, respectively. The exchange interaction between the NN and the radical‐cation is positive and ferromagnetic for **Ph2DTP‐Ph‐NN** and **MeSTh_2_DTP‐Ph‐NN**, whereas for **DTP‐Ph‐NN** it is negative indicating antiferromagnetic interaction. The spin densities were also calculated for both the neutral **R^1^**_**2**_ **DTP‐Ph‐NN** and charged (**R_2_DTP‐Ph‐NN)^.+^** molecules. The spin density is mostly localized on Ph‐NN for the neutral molecules while delocalized over the extended π‐unit for charged molecules in Figure 7 for **Ph_2_DTP‐Ph‐NN** (**Ph_2_DTP‐Ph‐NN**)**^.+^** and see in Figure S11 and S12 for **R_2_DTP‐PhNN**, **R^1^**_**2**_ **DTP‐Ph‐NN** and **(R^1^**_**2**_ **DTP‐Ph‐NN)^.+^** (R=H, and Th). The *J*_intra,calc_ is nearly five times higher for (**Ph_2_DTP‐Ph‐NN**)**^.+^** than for (**MeSTh_2_DTP‐Ph‐NN**)**^.+^** because the positive charges are distributed equally over the entire molecule for (**Ph_2_DTP‐Ph‐NN**)**^.+^**, whereas in **MeSTh_2_DTP‐Ph‐NN** the positive charge and the spin are better delocalized over the extended π‐unit and there is less spin on the central phenyl.

The *J*_intra,calc_ values indicate antiferromagnetic interaction for (**DTP‐PhNN**)**^.+^** but ferromagnetic interaction for (**Ph_2_DTP‐PhNN**)**^.+^** and (**MeSTh_2_DTP‐PhNN**)**^.+^**. To investigate this variation, vertical ionization potential (IP_ver_=*E*_cation_−*E*_neutral_) calculation was used. These (IP_ver_) calculations were performed by the DFT with BLYP, 6‐31G(d) basis set in the gas phase using the Gaussian 09 package (Figures S15 and S16, SI). The NN was deleted from the X‐ray structures for **Ph_2_‐DTP‐Ph** and **MeSTh_2_‐DTP‐Ph**, whereas for **DTP‐Ph** and **Ph‐NN**, optimized structures were used for calculation. The calculated IP_ver_ were 5.54 eV and 4.98 eV for **Ph_2_‐DTP** and **MeSTh_2_‐DTP‐Ph**, respectively. These are much lower than 6.24 eV of **NN‐Ph** (Figure S15). Therefore, neutral molecules of **Ph_2_DTP‐Ph‐NN**, **MeSTh_2_DTP‐Ph‐NN** are easily oxidized and form high spin ground state molecules. In case of **DTP‐Ph** the vertical ionization potential is IP_ver_=6.37 eV, which is higher than the IP_ver_=6.24 eV for phenyl nitronyl nitroxide radical (**Ph‐NN**, Figure S13). Consequently, in **DTP‐Ph‐NN^.+^** the nitroxide is charged with a low‐spin ground state and the triplet is much higher in energy.

As mentioned before, to generate a high‐spin biradical the arylamine of the DPT moiety must possess a lower oxidation potential than the phenyl‐NN moiety. The important factor now is that the energy difference between the HOMO and SOMO must be high enough. In the case of **DTP‐Ph‐NN**, the energy level of the SOMO is −5.18 eV and HOMO is −5.06 eV. Then, the energy difference between HOMO and SOMO is only (0.12 eV), whereas the HOMO of the phenyl nitronyl nitroxide (Ph‐NN) is −5.17 eV. It is very close to the SOMO of the **DTP‐Ph‐NN** (−5.18 eV). Therefore, we cannot determine very clearly that the removal of the first electron is either from the SOMO or HOMO for **DTP‐Ph‐NN** during oxidation. In other cases, the energy difference between the HOMO and SOMO were −0.39 and −1.04 for **Ph_2_DTP‐PhNN** and **MeSTh_2_DTP‐Ph‐NN**, respectively, which are sufficient to generate the cationic molecules.

## Conclusions

Three Ph‐NN substituted donor π‐core of (DTP) derivatives (**R_2_DTP‐Ph‐NN**) molecules were prepared and characterized. The molecular structures and packing of **Ph_2_DTP‐Ph‐NN** and **Th_2_DTP‐Ph‐NN** were examined by single‐crystal X‐ray structure analysis. The **Th_2_DTP‐Ph‐NN** is crystalized in the dimeric form with shorter intermolecular distance of N−O⋅⋅⋅C−Ph (3.31 Å), whereas **Ph_2_DTP‐Ph‐NN** crystallized as a monomeric structure with smaller intermolecular distance found between two oxygen atoms for N−O⋅⋅⋅N−O (3.24 Å) in molecular packing. Upon one‐electron oxidation, the (**R^1^**_**2**_ **DTP‐Ph‐NN**, R^1^=Ph and MeSTh)**^.+^** offered triplet ground state radical‐cationic high spin molecules. Although for (**DTP‐Ph‐NN**)**^.+^** the calculated magnetic interaction is antiferromagnetic between the NN and radical‐cation. Further charged molecules will be isolated and analyzed for magnetic properties by magnetic susceptibility. Syntheses of similar radical cationic molecules are under way. The **Ph_2_DTP‐Ph‐NN** and **MeSTh_2_DTP‐Ph‐NN** type molecules are suitable for spintronic and molecular magnetic materials applications.

## Experimental Section

Full experimental details can be found in the Supporting Information.

## Conflict of interest

The authors declare no conflict of interest.

## Supporting information

As a service to our authors and readers, this journal provides supporting information supplied by the authors. Such materials are peer reviewed and may be re‐organized for online delivery, but are not copy‐edited or typeset. Technical support issues arising from supporting information (other than missing files) should be addressed to the authors.

Supplementary
