# Supplementary material for: Dithienopyrrole Derivatives with Nitronyl Nitroxide Radicals and Their Oxidation to Cationic High‐Spin Molecules
Source: Chemistry. 2020 Feb 28;26(16):3626–32. doi: 10.1002/chem.201905734 (PMC7155055; doi:10.1002/chem.201905734)
Supplement: Supplementary file 1 — Supplementary [file CHEM-26-3626-s001.pdf]

CHEMISTRY
A European Journal

Supporting Information

Dithienopyrrole Derivatives with Nitronyl Nitroxide Radicals and Their Oxidation to Cationic High-Spin Molecules

Kubandiran Kolanji*[a, b] and Martin Baumgarten*[a]

chem_201905734_sm_miscellaneous_information.pdf

Table of Contents

1. Experimental Procedures……3
2. Syntheses……3
3. **Figure S1**: The known nitronyl nitroxide (NN) radical substituted radical cationic molecules……8
4. **Figure S2**: X–ray crystal structure of **Ph_2DTP-Ph-NN**, hydrogen atoms and PhCN are omitted for clarity……9
5. **Figure S3**: X–ray crystal structure of **MeSTh_2DTP–Ph-NN** (a) dimeric structure, (b) crystal packing (c) molecular structure **MeSTh_2DTP–Ph-NN –A** (d) molecular structure **MeSTh_2DTP-PhNN-B**, hydrogen atoms are omitted for clarity……9
6. **Figure S4**: Cyclic voltammograms of (a), (b) **DTP-Ph-NN**, (c) **Ph_2DTP-Ph-NN** in PhCN and (d) **MeSTh_2DTP-Ph-NN** in AcCN solution with 0.1M (n-C_4H_9)$_4$NBF_4 with scan rate = 0.1 V/s, Pt electrode versus ferrocene/ferrocenium (Fc/Fc^+)……10
7. **Figure S5**: UV–Vis absorption spectra of **Ph_2DTP–PhNN** and **MeSTh_2DTP-Ph-NN** in toluene (~10^{-6} M) solution. Inset zoom range (480-810 nm)……10
8. **Figure S6**: UV–Vis absorption spectra of **MeSTh_2DTP–PhNN** in toluene (~10^{-4} M) solution (solid green line) and its oxidation by addition of magic blue in CH_2Cl_2 at room temperature. Note: (broken lines) are formation of intermediates, (solid blue line) monoradical cation, and (solid red line) dication……11
9. **Figure S7**: EPR spectra of the (a) **DTP-Ph–NN** at rt, (d) for at 130K, (b) **Ph_2DTP-Ph-NN** at rt, (e) for at 130K, (c) **MeSTh_2DTP-Ph-NN** at rt, (f) for at 130K in toluene (~10^{-4} M) solution; (black) experimental, and (red) simulated……11
10. **Figure S8**: EPR spectra for during chemical oxidation reaction of (a) **DTP-Ph–NN,** (b), (c) **Ph_2DTP-Ph-NN**, (d) **MeSTh_2DTP-Ph-NN**, (e) (**Ph_2DTP-Ph-NN**)$^{+2}$, and (f) (**MeSTh_2DTP-Ph-NN**)$^{+2}$……11
11. **Figure S9:** Variable temperature EPR spectra's of the **Ph_2DTP-Ph-NN** and **MeSTh_2DTP-Ph-NN**……12
12. **Figure S10**: Variable temperature EPR spectra's of the **(Ph_2DTP-Ph-NN)$^{\cdot+}$** and (b) for 160K……12
13. DFT calculations……13
14. **Figure S11**: Optimized structures (a), (b), and (c), spin density distributions (d), (e) and (f) for **DTP-Ph-NN**, **Ph_2DTP-Ph-NN**, and **MeSTh_2DTP-Ph-NN**), respectively, calculated by DFT using ublyp/6-31g(d) basis set……13
15. **Figure S12**: Optimized structures (a), (b), and (c) and spin density distributions (d), (e) and (f) for the **(DTP-Ph-NN)$^{+\cdot}$**, **(Ph_2DTP-Ph-NN)$^{+\cdot}$**, and (**MeSTh_2DTP-Ph-NN**)$^{\cdot+}$ respectively. These were calculated by DFT using broken symmetry (BS) approach calculations……14
16. **Table S1**. The energy levels of the HOMO, LUMO, and SOMO in the eV for all the molecules of **R_2DTP-Ph-NN**……13
17. **Table S2**: The intra–molecular interaction DFT calculation details……14
18. **Figure S13**: (a) dimer structure of the (**Ph_2DTP-Ph-NN)$_2$** and (b) (**MeSTh_2DTP-Ph-NN)$_2$** for the inter molecular interaction calculation with spin distribution of the molecules……15
19. Table S3: The inter–molecular interaction DFT calculation details……15
20. **Figure S14**: Proposed one electron oxidation mechanism for **R_2DTP-Ph-NN**……15
21. **Figure S15**: Details of the vertical ionization potential calculation for (a) **DTP-Ph** (b) **DTP-Ph-NN** and (C) **Ph-NN**……16
22. **Figure S16**: Details of the vertical ionization potential calculation **Ph_2DTP-Ph** and (b) **MeSTh_2DTP-Ph-NN**……16
23. Crystal data for **Ph_2DTP-Ph-NN**……17
24. Crystal data for **MeSTh_2DTP-Ph-NN**……18
25. **Figures S15-S27:** NMR spectra……19-26
26. **References S1**……27

Experimental Procedures

Materials and methods: Oven-dried glassware were used for all the reactions and experiments. All manipulations were performed under a dry argon atmosphere using a standard technique. All reagents and chemicals were purchased from commercial sources and used as received, unless otherwise specified.

Column chromatography: The column chromatography was performed using by silica gel (60–120, 100–200 and 230–400 mesh). For thin layer chromatography, aluminium sheets pre-coated with silica gel (Merck, Kieselgel 60, F254) were employed.

NMR spectroscopy: Proton (1H NMR) and carbon (13C NMR) nuclear magnetic resonance spectra were recorded using 250, 300, 500, or 700 MHz Bruker spectrometers. Chemical shifts are reported for 1H NMR and 13C NMR relative to residual proton or carbon resonances of the deuterated solvents.

Mass spectrometry: The high-resolution electrospray ionization mass spectrometry (HR-ESI-MS) was performed at the Institute for Organic Chemistry, Johannes-Gutenberg-University of Mainz, on an ESI-Q-TOF system (maXis, Bruker Daltonics, Germany).

EPR spectroscopy: EPR spectra were recorded in diluted and argon-purged solutions of toluene or DCM with the concentration of 10^{-4} molar unless otherwise stated by using a using a Bruker EMX-plus spectrometer equipped with the gauss meter and a variable-temperature control continuous-flow-N2 cryostat (Bruker B-VT 2000). The g-factor corrections were obtained by using DPPH (g = 2.0037) as standard. The spectral simulation carried out using Bruker Win EPR Sim Fonia software.

UV–Vis absorption spectroscopy: UV-Vis spectra were recorded in toluene or DCM with Perkin Elmer Spectrometer (UV/Vis/NIR Lambda 900) by using 1 cm optical–path quartz cell at room temperature. The baseline was corrected by subtracting a measurement of the cuvette filled with pure solvent used for the measurement.

Single-crystal X-ray measurements: The single crystal X-ray crystallographic data were collected on a STOE IPDS 2T diffractometer using a graphite monochromator Mo–Kα as a radiation source. The structures were solved by direct methods (SIR-2004) and refined by SHELXL–2014 (full matrix).

Cyclic voltammetry (CV): CV measurements were carried out on a computer-controlled GSTAT12 in a three-electrode cell in anhydrous PhCN, acetonitrile or DCM solution of n-Bu4NPF6 (0.1 M) with a scan rate of 100 mV/s at room temperature. A Pt wire, a silver wire, and a glassy carbon electrode were used as the counter electrode, the reference electrode, and the working electrode, respectively. The oxidative process with half-wave potentials are summarized versus ferrocene/ferrocenium (Fc/Fc^+).

Syntheses

2-(4-Nitrophenyl)-1,3-dioxolane.[1]

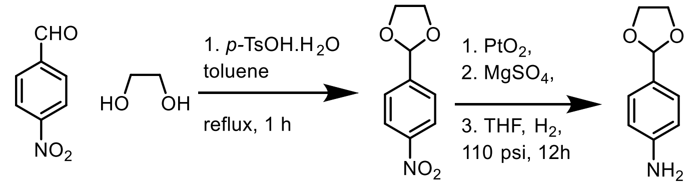

1

The 4-nitrobenzaldehyde (3.30 g, 21.83 mmol) and p-toluenesulfonic acid monohydrate (76.5 mg, 0.402 mmol) were dissolved (75 mL) toluene in 250 mL round bottom flask. Then ethylene glycol (2.5 mL) was added, and the solution was refluxed with a Dean-Stark trap to azeotropically remove water. After 1 hour, the solution was allowed to cool to ambient temperature and 100 mL of Et_2O was added. The solution was washed twice with saturated $NaHCO_3$ solution and then with saturated NaCl solution. The solution was dried over $MgSO_4$ and evaporated to yield a pale yellow solid (4 g, 94% yield). ^{1}H NMR (250 MHz, CD_2Cl_2, δ, ppm): 8.20 (d, 2H), 7.65 (d, 2H), 5.87 (s, 1H), 4.07 (m, 4H).

4-(1,3-Dioxolan-2-yl)aniline (1).[2] A pressure vessel was charged with 2-(4-nitrophenyl)-1,3- dioxolane (2.00 g, 10.25 mmol), PtO_2 (0.1 g, 0.40 mmol), $MgSO_4$ (2.5 g, 20.75 mmol), and THF (15 mL). The reaction vessel was purged with N_2 and subsequently charged with H_2 (110 psi). The reaction was stirred for 12 hr at room temperature while maintaining the H_2 pressure by periodically recharging the vessel. The reaction mixture was filtered through a fritted glass funnel, and washed with dry THF. The filtrates were combined and volatile fractions were removed to afford 1 (1.6 g, > 98%). ^{1}H NMR (250 MHz, CD_2Cl_2, δ, ppm): 7.22 (d, 2H), 6.64 (d, 2H), 5.63 (s, 1H), 4.03 (m, 4H).

3,3'-diiodo-2,2'-bithiophene (2).[3]

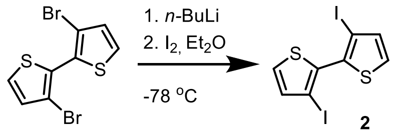

A 3,3'-dibromo-2,2'-bithiophene (3.01 g, 16.6 mmol) was dissolved in anhydrous Et_2O (90 mL) under a N_2-atmosphere In a flame-dried round bottom flask. Moreover, *n*-BuLi (1.6 M in hexanes, 13 mL, 18.56 mmol, 2.2 equiv.) was added dropwise at –78 °C. The reaction mixture was stirred about 1 h. A solution of I_2 (5.17 g, 18.56 mmol, 2.2 equiv.) in Et_2O (18 mL) was added dropwise, the mixture was warmed to r.t. and stirred for another hour. The reaction was quenched by addition of saturated $Na_2S_2O_3$ (60 mL) and the layers were separated. The aqueous layer was extracted with Et_2O (2×60 mL). The combined organic layer was washed with brine, dried over $MgSO_4$ and the solvent was evaporated. The residue was recrystallized from hexanes/toluene 2:1. Molecule **2** was isolated as an off-white crystalline solid (2.7 g, 70%).^{1}H NMR (300 MHz, CD_2Cl_2, δ, ppm): 7.46 (d, 2H), 7.18 (d, 2H).

4-(4H-dithieno[3,2-b:2',3'-d]pyrrol-4-yl)benzaldehyde, (3).[4]

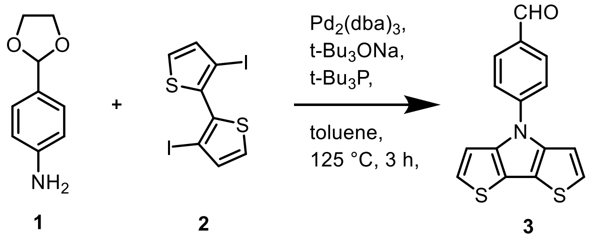

Under nitrogen atmosphere, the 500 mL round bottom flask connected with condenser and charged with 3,3'-diiodo-2,2'-bithiophene, (2), (8.0 g, 19.14 mmol), $Pd_2(dba)_3$ (0.887 g, 0.97 mmol) and sodium *tert*-butoxide (2.23 g, 23.20 mmol) in toluene (200 mL), and (2.9 mL) $P(^tBu)_3$ in 1 M toluene solution was added. Then, the mixture was stirred at 35 °C for 15 mints. Further, freshly prepared 4-(1,3-dioxolan-2-yl)aniline, (1)[2] (4.5 g, 27.24 mmol) in THF was added and the mixture was refluxed at 125 °C another 2.5 h and the reaction mixture was cooled to room temperature. The reaction mixture was filtered, extracted with diethyl ether, washed with brine solution and concentered. The residue was dissolved in THF and 1 M HCl was added and then the mixture was stirred for 2 h. Further, the reaction mixture was extracted with diethyl ether washed with brine solution. The crude mixture purified by column chromatography, yielded yellow solid (2.5 g, 46 %). ^{1}H NMR (250 MHz, CD_2Cl_2, δ, ppm): 10.03 (s, 1H), 8.06 (d, J = 7.5 Hz, 2H, Ph-H), 7.79 (d, J = 7.5 Hz, 2H, Ph-H), 7.27 (s, 4H, Th-H). ^{13}C NMR (62.90 MHz,CD_2Cl_2, δ, ppm): 191.3, 145.2, 143.9, 134.1, 132.0, 124.7, 122.5, 118.8, 113.1. HRMS m/z: calcd for $C_{15}H_9NOS_2$, 283.0126, ion formula for $[M+H]^+$, $C_{15}H_{10}NOS_2$, 284.0126; found, 284.0188.

2-(4-(4H-Dithieno[3,2-b:2',3'-d]pyrrol-4-yl)phenyl)-4,4,5,5-tetramethylimidazolidine-1,3-diol (4).

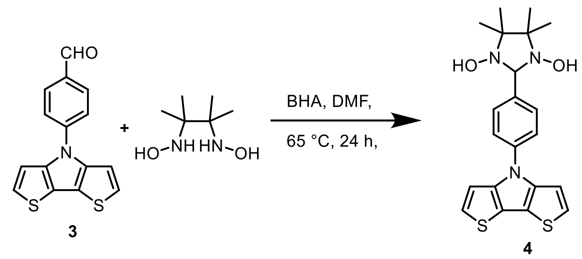

A mixture of 3 (0.290 g, 1.02 mmol) and 2,3-bis(hydroxylamino)-2,3-dimethylbutane (0.18 g, 1.22 mmol) in 15 mL of DMF was stirred at 65 C, for 24 h. The solution was concentrated and obtained residue was washed with ethyl acetate, obtained 4 as yellow solid (0.22 g, 52%). ^{1}H NMR (300 MHz, DMSO-d^{6}, δ ppm): 7.84 (s, 2 H, N-OH), 7.66 (m, 4 H), 7.47 (d, 2 H), 7.29 (d, 2 H), 4.58 (s, 1 H), 1.10 (s, 12 H. ^{13}C NMR (75 MHz, DMSO-d^{6}, δ ppm): 143.4, 139.9, 138.1, 130.2, 124.7, 121.4, 116.1, 112.6, 90.1, 66.2, 24.5, 17.2; ESI calculated for $C_{21}H_{23}N_3O_2S_2$, 413.12, found: 414.6, $[M+H]^+$.

Synthesis of the DTP-Ph-NN.

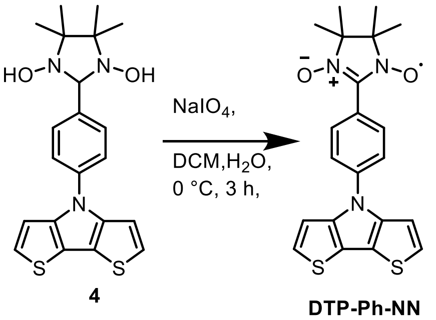

The compound 4 (0.101 g, 0.244 mmol) was dissolved in DCM and water, then $NaIO_4$ (0.057 g, 0. 267 mmol) in water was added at 0 C. The reaction mixture was stirred for 3 h at 0 C, mean time reaction was monitored by TLC. After the reaction was completed green organic phase was extracted by dichloromethane, and then washed with water and brine. The organic phase dried over $MgSO_4$ and concentrated. The residue was chromatographed over silica gel using DCM as eluent to obtain green product, 62 mg, 62 % yield; EPR (298 K, ~10^{-4} M in toluene): five lines, g_{iso} = 2.0066, $a_{N1}/2$ = 0.37 ±0.002 *mT*. HRMS m/z: calcd for $C_{15}H_9NOS_2$, 283.0126, ion formula for $[M+H]^+$, $C_{15}H_{10}NOS_2$, 284.0126; found, 284.0188.HRMS m/z: calcd for $C_{21}H_{20}N_3O_2S_2$, 410.0997, ion formula for $[M+H]^+$, $C_{21}H_{21}N_3O_2S_2$, 411.1070; found, 411.1069.

Synthesis of 4-(4-(1,3-bis((tert-butyldimethylsilyl)oxy)-4,4,5,5-tetramethylimidazolidin-2-yl)phenyl)-4H-dithieno[3,2-b:2',3'-d]pyrrole, (5).

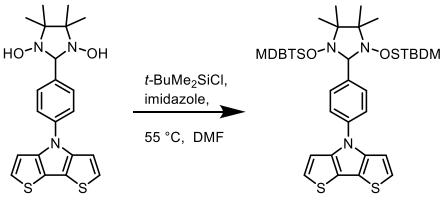

A mixture of 4, (1.01 g, 2.44 mmol), *t*-butyldimethylsilyl chloride 1.90, 12.61 mmol), and imidazole (1.66 g, 24.42 mmol) taken in 100 mL Schlenk flask and dissolved in DMF (30 mL) and the mixture was stirred for 24 h at 55 °C under argon atmosphere. The solvent was removed under reduced pressure, and then the crude product was extracted with ether and washed with water. The ether layer was dried over magnesium sulfate and concentrated under reduced pressure. The residue was chromatographed on silica gel with hexane as the eluent to give **5**, colorless solid (1.3 g, 83%).^{1}H NMR (CD_2Cl_2, 250 MHz): δ, 7.54 (m, 4 H), 7.20 (d, 2 H), 7.12 (d, 2 H), 4.71 (s, 1 H), 1.20 (s, 12 H), 0.81 (s, 18 H), 0.01 (s, 6 H), -0.76 (s, 6 H). ^{13}C NMR (75 MHz, CD_2Cl_2,) δ, 144.8, 139.9, 132.9, 132.9, 123.9, 122.7, 116.9, 112.7, 89.9, 68.5, 27.4, 18.4, 17.5, -3.5, -4.7; HRMS m/z: calcd. for $C_{33}H_{51}N_3O_2SSi_2$, 641.2961, ion formula for $[M+H]^+$, $C_{33}H_{52}N_3O_2SSi_2$, 642.3034; found, 642.3037.

Synthesis of 6.

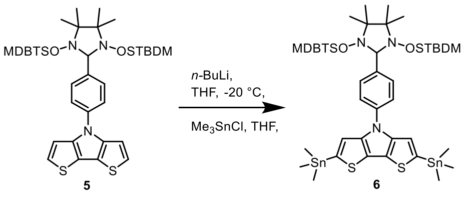

The compound **5** (1.02 g, 1.588 mmol) was dissolved in dry hexane (50 mL) and the solution was cooled to 0°C. Then tetramethylethylenediamine (TMEDA) (0.553 g, 0.72 mL, 4.759 mmol) was added, followed by BuLi (1.6 M in hexanes, 2.2 mL, 3.49 mmol) and the mixture stirred for 2 h at 0°C, Me_3SnCl (0.799 g, 3.97 mmol) was then added and the solution stirred overnight at rt. This mixture was poured over Et_3N-treated silica gel, filtered, and rinsed with hexane. The filtrate was concentrated via rotary evaporation and then dried overnight. The product was stored under N_2 in the freezer until further use. colourless solid (1.4 g, 91 %). ^1H NMR (CD_2Cl_2, 700 MHz): δ, 7.56 (d, 2 H), 7.52 (d, 2 H), 7.11 (s, 2 H), 4.72 (s, 1 H), 1.20 (s, 12 H), 0.82 (s, 18 H), 0.39 (s, 18 H), 0.02 (s, 6 H), -0.74 (s, 6 H). ^{13}C NMR (176 MHz, CD_2Cl_2,) δ, 147.7, 140.4, 140.1, 137.3, 123.6, 122.9, 122.5, 119.6, 93.02, 68.5, 26.5, 24.5, 17.4, -4.1 (Si(Me), -7.9 (Sn(Me3); HRMS m/z: calcd for $C_{39}H_{67}N_3O_2S_2Si_2Sn_2$, 969.2257, ion formula for $[M+H]^+$, $C_{39}H_{68}N_3O_2S_2Si_2Sn_2$ 970.2335; found, 970.2.

2-Bromo-5-(methylthiol)thiophene.[5]

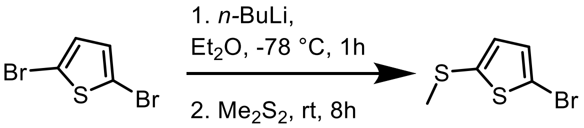

A 2,5-Dibromothiophene (4.0 g, 16.53 mmol) was dissolved in diethyl ether (30 mL) and then cooled to –78°C. A solution of *n*-butyl lithium (1.6 M in hexanes, (11.8 mL, 19.01 mmol, 1.15 eq)) was slowly added while maintaining the temperature at less than –65°C. After complete mono-exchange, a solution of dimethyldisulfide (1.4 mL, 16.53, 1 eq.) in diethyl ether (2.0 mL) was added and the cooling bath was removed while stirring, allowing the mixture to warm up to ambient temperature. Then the mixture was diluted with water (50.0 mL) and organic layer was separated by separating funnel. The organic layer was washed with water (2x50 mL), and brine solution (1x50 mL). Then, dried over $MgSO_4$, and concentrated to form a black residue. The residue was passed through a silica gel plug and eluted with hexanes. Evaporation of the organics afforded the desired compound as a tan oil (3.4 g, 98 % yield). ^1H NMR (CD_2Cl_2, 250 MHz): δ, 6.91 (s, 2 H), 2.472 (s, 6 H).

General procedure for Stille Coupling.

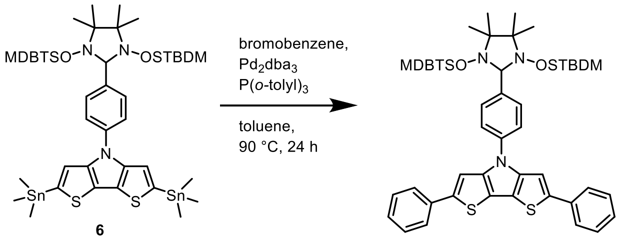

6

7a

The desired intermediate **6** (0.1 mmol, 1 eq) and bromobenzene or 2–bromo–5–(methylthio)thiophene (0.25 mmol, 2.5 eq) were taken in 50 mL a Schlenk tube. The mixture was evacuated and backfilled with Ar_2 three times. And then $Pd_2(dba)_3$ (0.01 mmol, 0.1 eq), $P(o\text{-tolyl})_3$ (0.03 mmol, 0.3 eq), and degassed toluene (20 mL) were added. The reaction was heated under stirring at 60 °C until completion (ca. 30 h) providing 7a or 7b, respectively.

7a, 62 %,: ^1H NMR (CD_2Cl_2, 700 MHz): δ, 7.65 (d, 4 H), 7.62 (d, 2 H), 7.59 (d, 2 H), 7.40 (t, 4 H), 7.37 (s, 2 H), 7.29 (t, 2 H), 4.75 (s, 1 H), 1.22 (s, 12 H), 0.84 (s, 18 H), 0.04 (s, 6 H), -0.70 (s, 6 H). ^{13}C NMR (176 MHz, CD_2Cl_2, δ, ppm): 147.8, 144.9, 143.0, 139.6, 135.8, 129.5, 137.9, 125.8, 123.1, 122.5, 116.6, 108.6, 93.6, 68.5, 26.7, 24.5, 17.1, -4.1; HRMS m/z: calcd for $C_{45}H_{59}N_3O_2S_2Si_2$, 793.3587, ion formula for $[M+H]^+$, $C_{45}H_{60}N_3O_2S_2Si_2$, 794.3665; found, 794.3.

7b, 69 %:

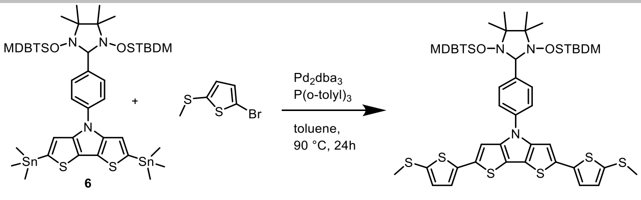

^{1}H NMR (CD_2Cl_2, 300 MHz): δ, 7.61 (d, 2 H), 7.52 (d, 2 H), 7.11 (s, 2 H), 7.06 (d, 2 H), 6.99 (d, 2 H), 4.75 (s, 1 H), 2.52 (s, 6 H), 1.21 (s, 12 H), 0.83 (s, 18 H), 0.03 (s, 6 H), -0.71 (s, 6 H). ^{13}C NMR (176 MHz, CD_2Cl_2, δ, ppm): 147.8, 144.68, 140.9, 139.3, 136.9, 135.7, 132.5, 132.2, 123.8, 123.1, 116.1, 108.9, 93.4, 68.6, 30.26, 25.5, 22.4, 18.4, -3.46; HRMS calculated for $C_{43}H_{60}N_3O_2S_6$, 898.2543, found: 898.2522, $[M+H]^+$.

General procedure for preparation for Ph$_2$DTP-Ph-NN and MeSTh$_2$DTP-Ph-NN.

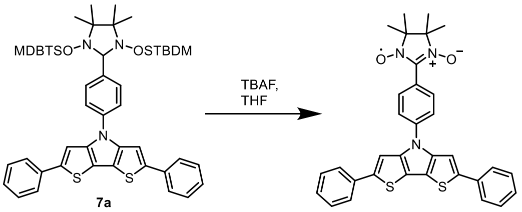

The compound 7a, or 7b (1 eq.) was dissolved in THF and TBAF (2 eq.) was added to the reaction mixture at room temperature. The reaction mixture was stirred for 6 h, mean time reaction was monitored by TLC. After the reaction was completed, a green solution was concentrated. The residue was chromatographed over silica gel using DCM:MeOH (99:1) as eluent.

Ph$_2$DTP-Ph-NN. Yield (72 %), EPR (298 K, ~10^{-4} M in toluene): five lines, g_{iso} = 2.0066, $a_{N1}/2$ = 0.37 *mT*. HRMS m/z: calcd for $C_{33}H_{28}N_3O_2S_2$, 562.1623, ion formula for $[M+H]^+$, $C_{33}H_{29}N_3O_2S_2$, 563.1696, found, 563.1692.

Synthesis of the MeSTh$_2$DTP-Ph-NN.

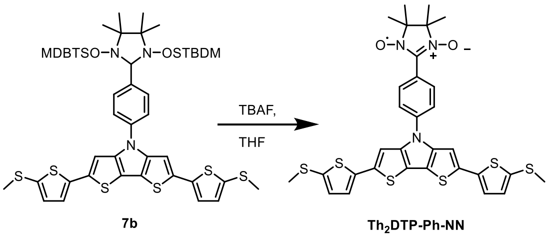

Obtain green crystalline product yield (69 %), EPR (298 K, ~10^{-4} M in toluene): five lines, g_{iso} = 2.0066, $a_{N1}/2$ = 0.37 *mT*. HRMS m/z: calcd for $C_{31}H_{28}N_3O_2S_6$, 666.9440, ion formula for $[M+H]^+$, $C_{31}H_{29}N_3O_2S_6$, 667.0584, found, 667.0.

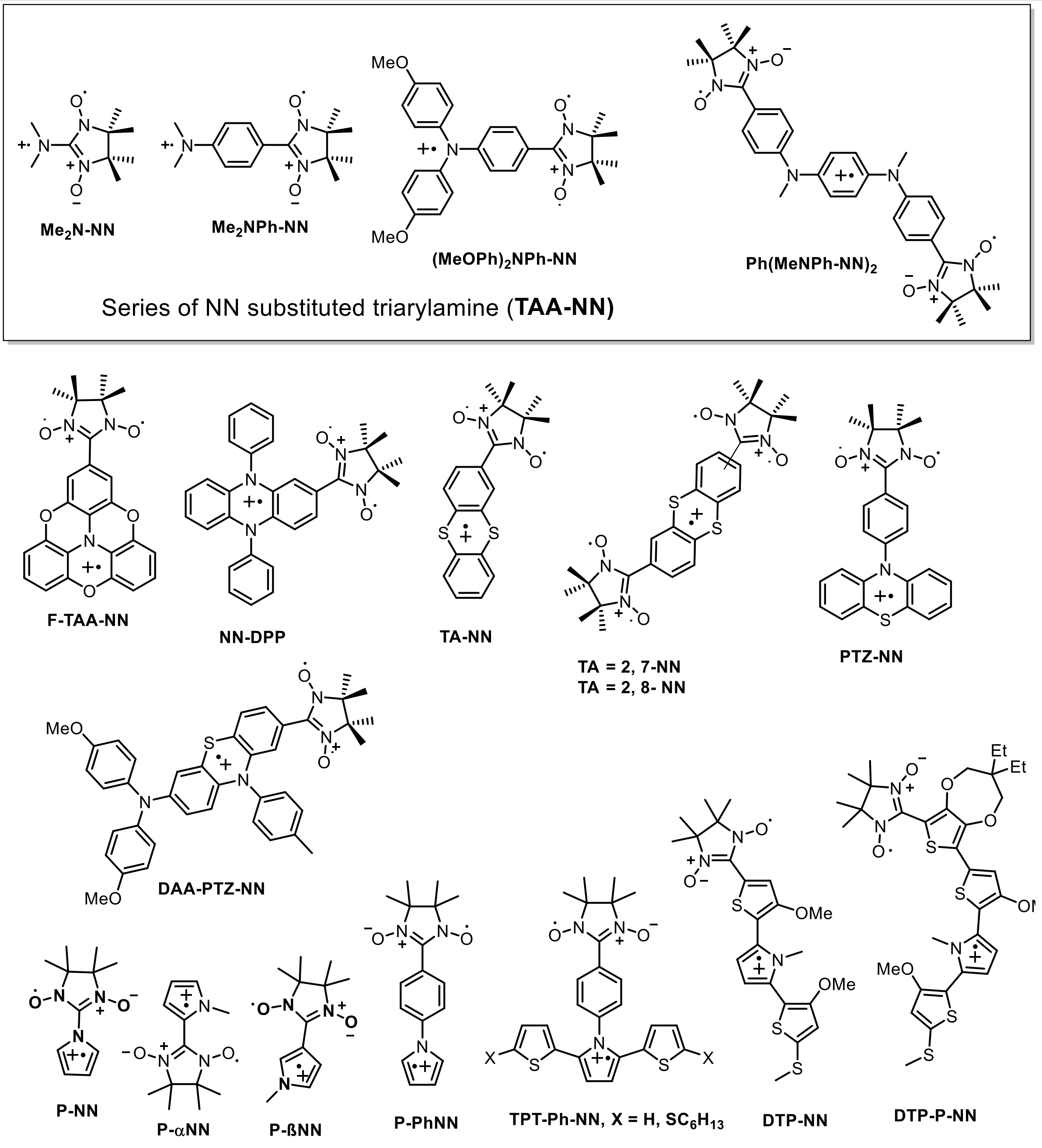

Figure S1: The known nitronyl nitroxide (NN) radical substituted radical cationic molecules.

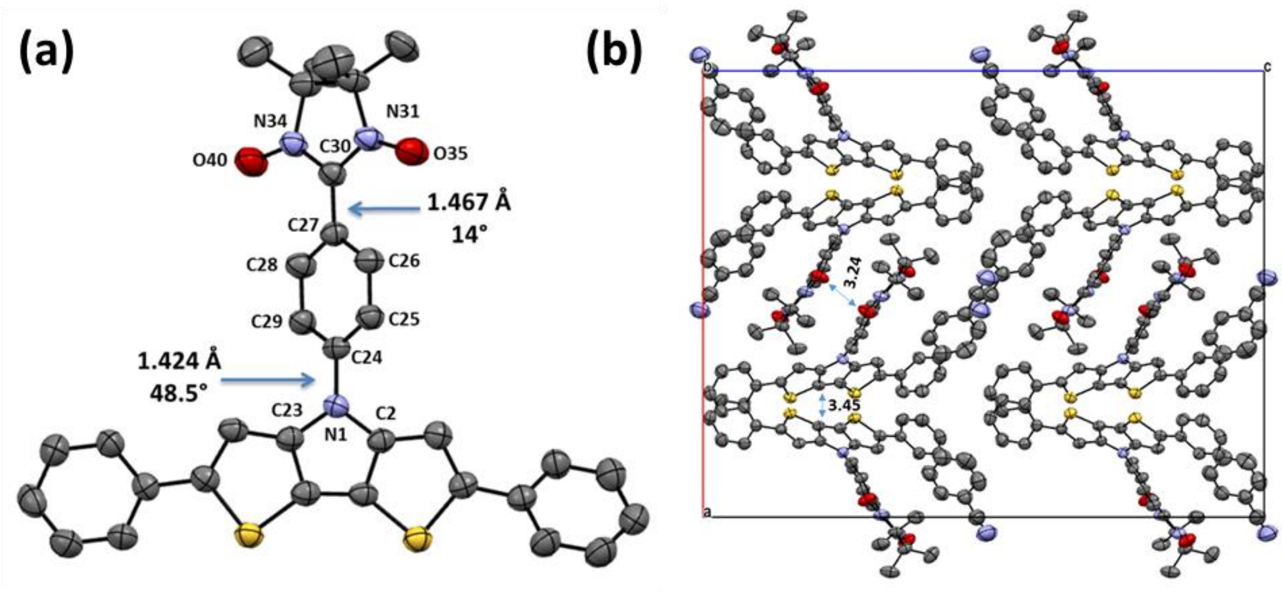

Figure S2: X–ray crystal structure of **Ph$_2$DTP-Ph-NN**, hydrogen atoms and PhCN are omitted for clarity.

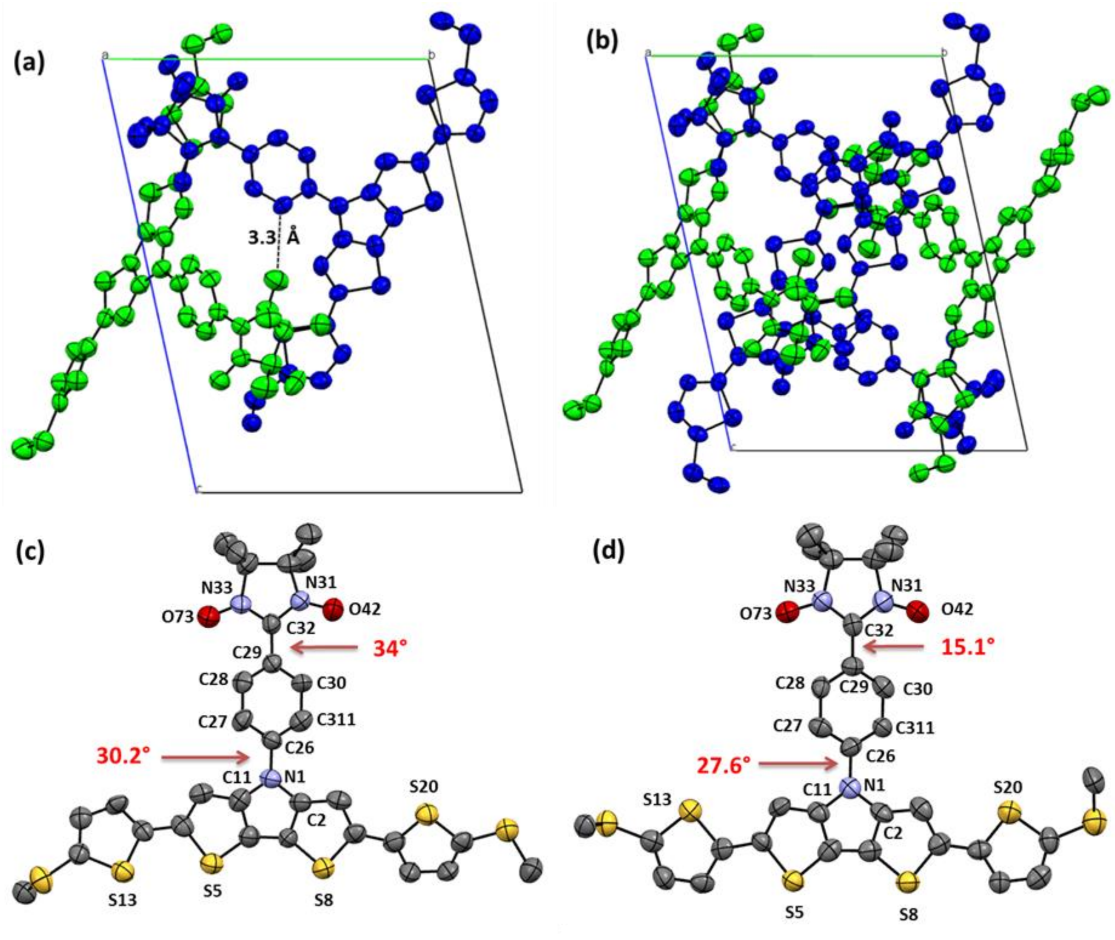

Figure S3: X–ray crystal structure of **MeSTh$_2$DTP-Ph-NN** (a) dimeric structure, (b) crystal packing (c) molecular structure **MeSTh$_2$DTP-Ph-NN-A** (d) molecular structure **MeSTh$_2$DTP-Ph-NN-B**, hydrogen atoms are omitted for clarity.

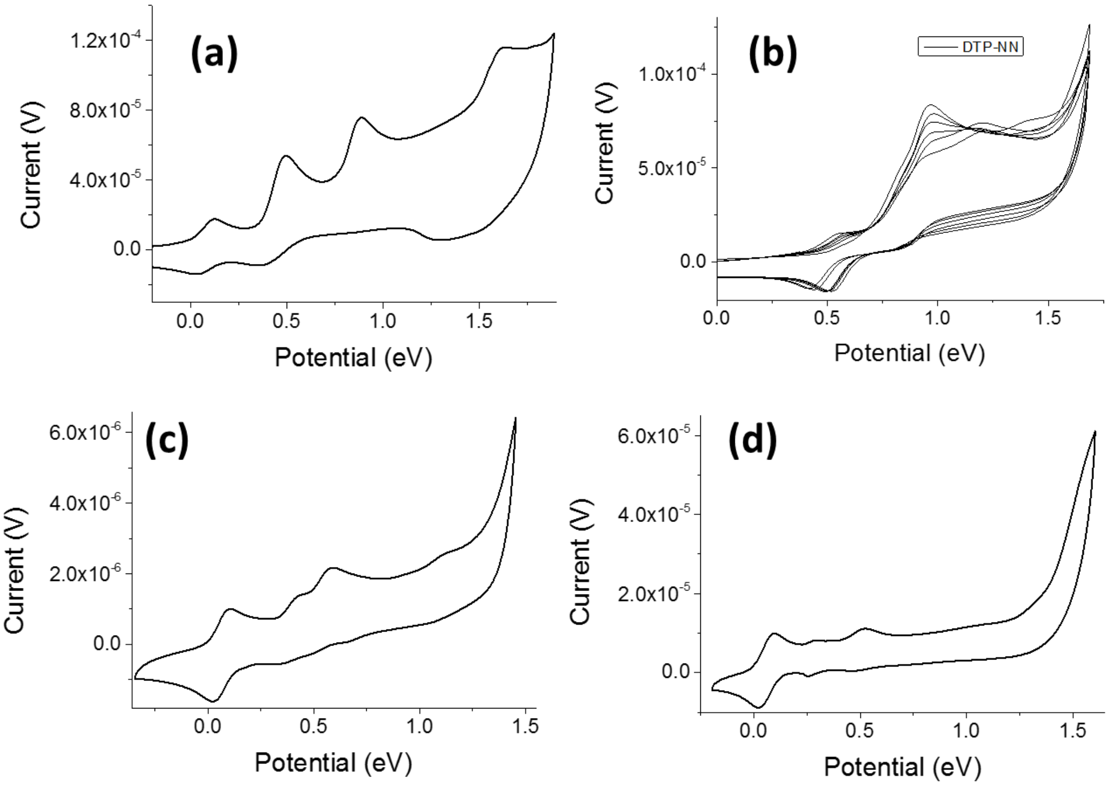

Figure S4: Cyclic voltammograms of (a), (b) **DTP-Ph-NN**, (c) **Ph_2DTP-Ph-NN** in PhCN and (d) **MeSTh_2DTP-Ph-NN** in AcCN solution with 0.1M $(n\text{-}C_4H_9)_4NBF_4$ with scan rate = 0.1 V/s, Pt electrode and referenced to versus ferrocene/ferrocenium (Fc/Fc^+).

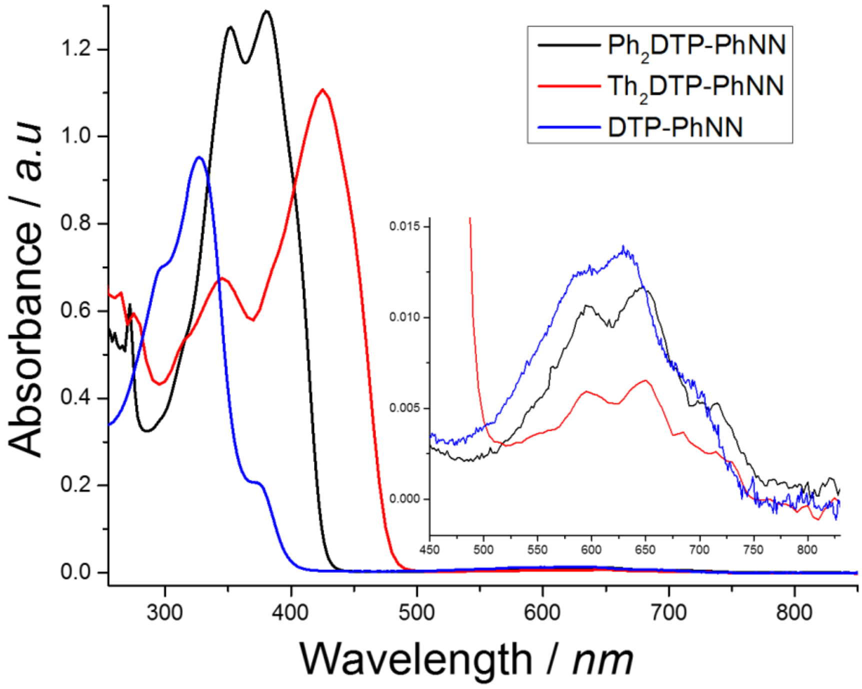

Figure S5: UV–Vis absorption spectra of **Ph_2DTP-Ph-NN** and **MeSTh_2DTP-Ph-NN** in toluene (~10^{-6} M) solution. Inset zoom range (480-810 nm).

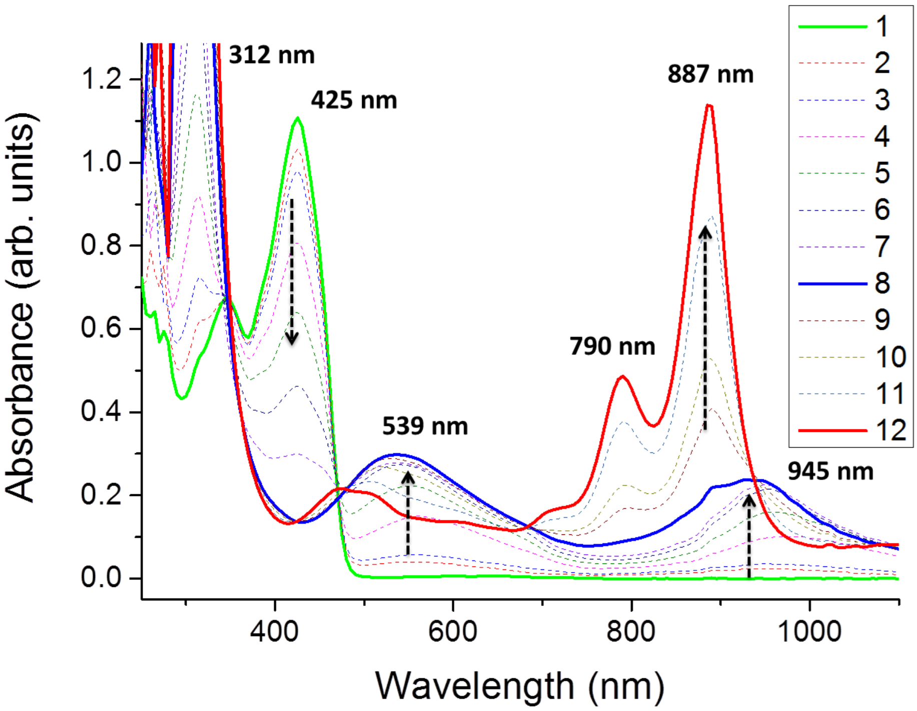

Figure S6. UV–Vis absorption spectra of **MeSTh$_2$DTP-Ph-NN** in toluene (~10^{-4} M) solution (solid green line) and its oxidation by addition of magic blue in CH_2Cl_2 at room temperature. Note: (broken lines) are formation of intermediates, (solid blue line) monoradical cation, and (solid red line) dication.

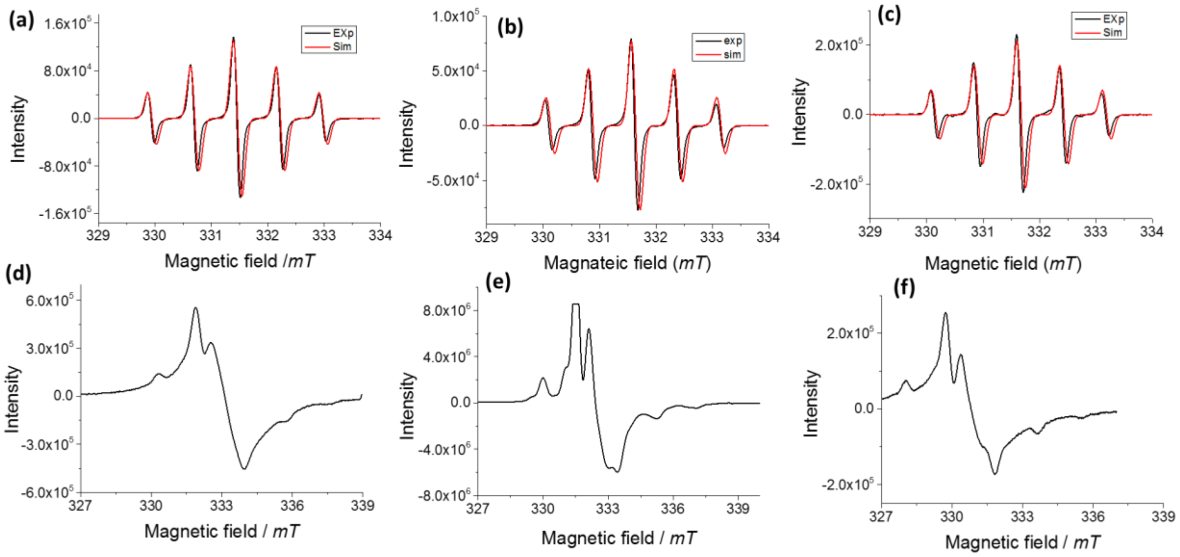

Figure S7: EPR spectra of the (a) **DTP-Ph-NN** at rt, (d) for at 130K, (b) **Ph$_2$DTP-Ph-NN** at rt, (e) for at 130K, (c) **MeSTh$_2$DTP-Ph-NN** at rt, (f) for at 130K in toluene (~10^{-4} M) solution; (black) experimental, and **(**red) simulated.

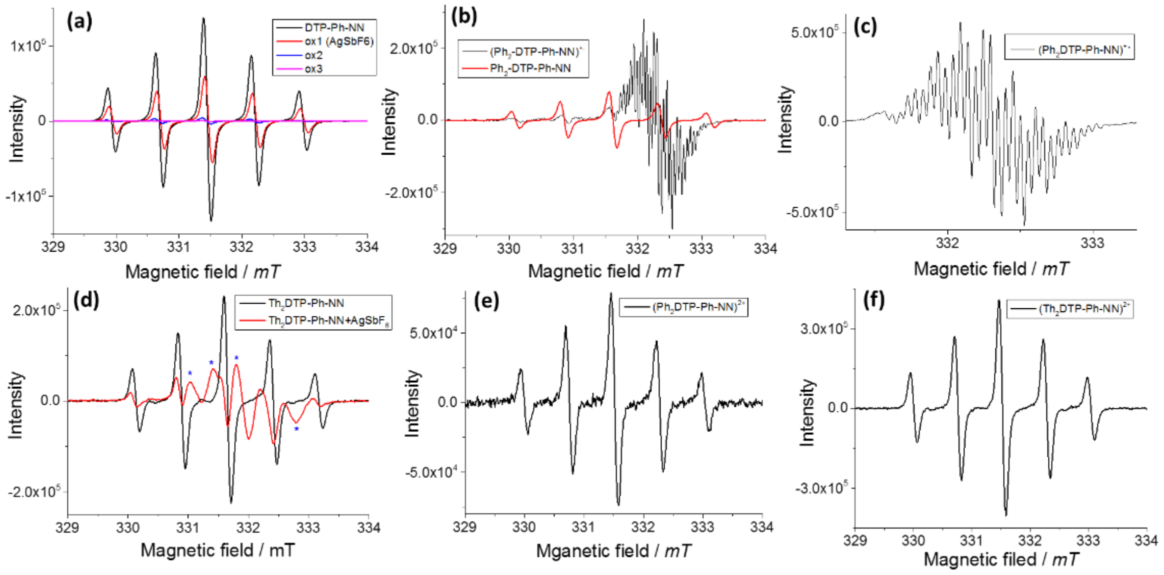

Figure S8: EPR spectra for during chemical oxidation reaction of (a) **DTP-Ph-NN,** (b), (c) **Ph_2DTP-Ph-NN**, (d) **MeSTh$_2$DTP-Ph-NN**, (e) **(Ph_2DTP-Ph-NN)$^{+2}$**, and (f) **(MeSTh$_2$DTP-Ph-NN)$^{+2}$**.

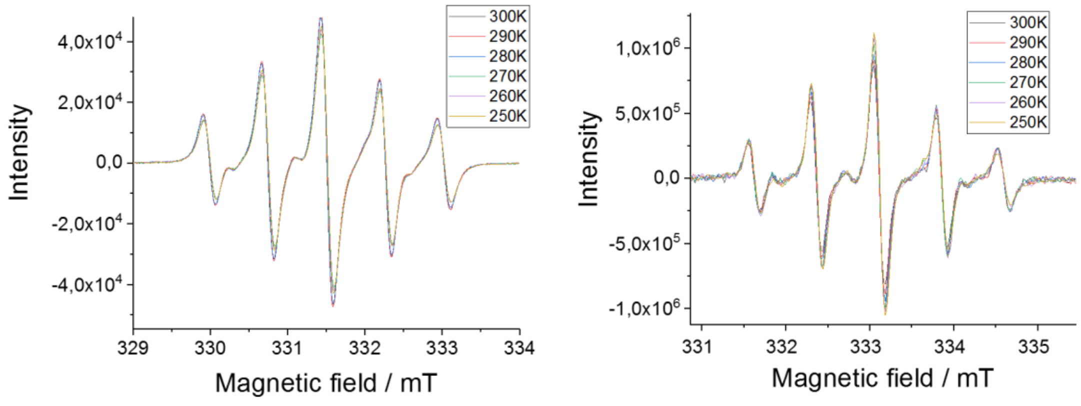

Figure S9: Variable temperature EPR spectra's of the **Ph_2DTP-Ph-NN and MeSTh$_2$DTP-Ph-NN**

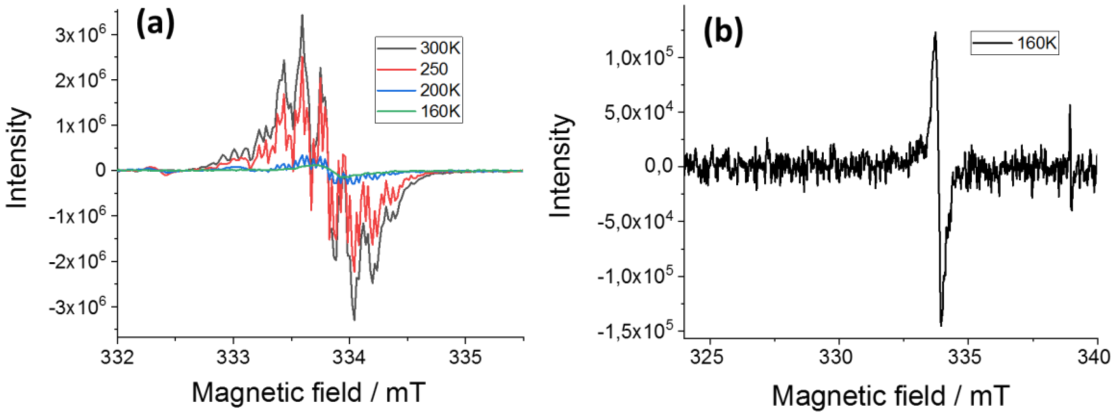

Figure S10: Variable temperature EPR spectra's of the (**Ph_2DTP-Ph-NN)$^{\cdot+}$** and (b) for 160K

DFT calculations

The computations were carried out to understand the electronic structure of the molecules. All the DFT calculations were performed using the Gaussian09 package.[6] The full geometry optimizations were carried out by UB3LYP/6–31G(d) level for all the neutral radical molecules. The optimized structures and spin density distribution of the neutral radical molecules of **DTP-Ph-NN**, **Ph_2DTP-Ph-NN**, and **MeSTh$_2$DTP-Ph-NN** are shown in Figure S9. The bond distance between NN and Ph unit is 1.461 Å and between Ph and DTP–backbone is 1.412, these values are same for all the derivatives. The torsion between NN and its attached phenyl is slightly varies for different derivatives such as 7.2°, 1.4°, and 1.7°, similarly between phenyl and DTP–backbone also varies as 39.9, 48.9, 41.1 for **DTP-Ph-NN**, **Ph_2DTP-Ph-NN**, and **MeSTh$_2$DTP-Ph-NN** respectively. In order to rationalize the ground state spin multiplicity of the molecules, the optimization was carried out for one electron oxidized molecular structures of **(DTP-Ph-NN)$^{\bullet+}$**, **(Ph_2DTP-Ph-NN)$^{\bullet}$+** and **(MeSTh$_2$DTP–Ph-NN)$^{\bullet}$+** and the structures are shown in Figure S10. The cation–diradicals have two singly occupied molecular orbitals, SOMO and SOMO', the latter resulting from the HOMO through one electron oxidation.

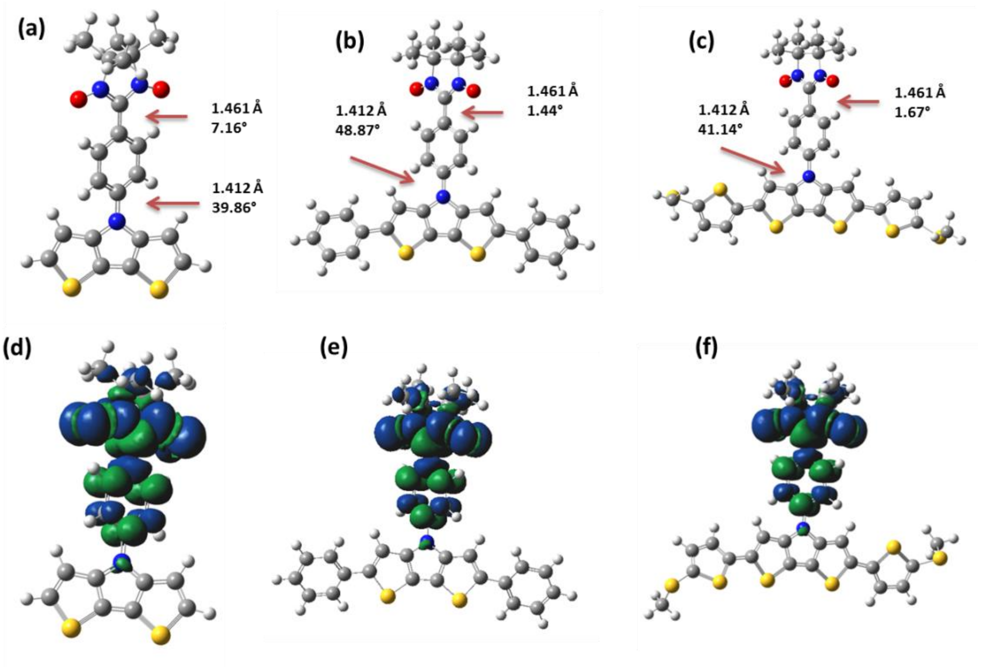

Figure S11: Optimized structures (a), (b), and (c), spin density distributions (d), (e) and (f) for **DTP-Ph-NN, Ph_2DTP-Ph-NN,** and **MeSTh$_2$DTP-Ph-NN),** respectively, calculated by DFT using ublyp/6-31g(d) basis set.

Table S1. The energy levels of the HOMO, LUMO, and SOMO in the eV for all the molecules of **R_2DTP-Ph-NN**

	HOMO	SOMO	LUMO	HF
**DTP-Ph-NN**	–5.18	–5.06	–1.63	–1923.2130285
**Ph_2DTP-Ph-NN**	–5.19	–4.80	–4.80	–2385.3248196
**MeSTh$_2$DTP-Ph-NN**	–5.26	–4.22	–5.26	–3901.8373782

The energy levels were calculated by DFT using ub3lyp/6-31g(d) basis set.

From optimized structure, the spin density distribution was calculated by DFT using ublyp/6-31g(d) basis set. For neutral radical molecules of **Ph_2DTP-Ph-NN, MeSTh$_2$DTP-Ph-NN** and **DTP-Ph-NN**, the spin densities are mostly distributed on NN unit and Ph part (Figure S9d–f). For the one electron oxidized molecules, the NN radical spin are more distributed on phenyl unit while extending the

DTP π–bridge the spin have decreased in the Ph part. The positive charges are distributed nearly equally over the entire molecule for **Ph_2DTP-Ph-NN** and **Th_2DTP-Ph-NN** while the positive charge and the spin may be better delocalized over the extended π–unit. Less spin is on central phenyl for (Figure S10d–f). Further, brokensymmetry (BS) approach calculations were applied to calculate exchange interaction because B3LYP hybrid function was over estimated.[7] The spin contaminations errors were corrected by Heisenberg−Dirac−Van Vleck (HDVV) Hamiltonian.[8] The J_{intra}/k_B was calculated from the optimized structure. The singlet and triplet energy were calculated by UBLYP hybrid function using 6–31G(d) basic set (to avoid Hartree-Fock contamination).

$$H = –2J_{12}\ S_1S_2,$$

S1 and S2 are the spin angular momentum operators.

Exchange interaction, $J = (E(BS) – E(T)) / (S^2(T) –S^2(BS))$

where, E(BS) is the energy of the broken-symmetry (BS) approach, this approach uses the guess=mix keyword to build up, as initial guess, a 1:1 mixture of singlet and triplet states with $S^2 = 1$, E(T) is the energy of the triplet state with $S^2(T) = 2$, and S^2 are the eigen values of the spin operator for these states.

Thus direct exchange yields $J = E(BS) – E(T)$

Table S2: Calculated triplet–, singlet–energy and exchange interactions (J_{intra}/kB) of the donor radicals cation (**R_2DTP–Ph–NN)**$^{•+}$

	HF (triplet)	HF (singlet)	J_{intra}/k_B (K)
**(DTP–PhNN)**$^{•+}$	–1922.5149694	–1922.528097	–3580
**(Ph_2DTP–PhNN)**$^{•+}$	–2384.45220	–2384.4363728	+5000
**(MeSTh_2DTP–PhNN)**$^{•+}$	–3900.9501544	–3900.947097	+ 965.4

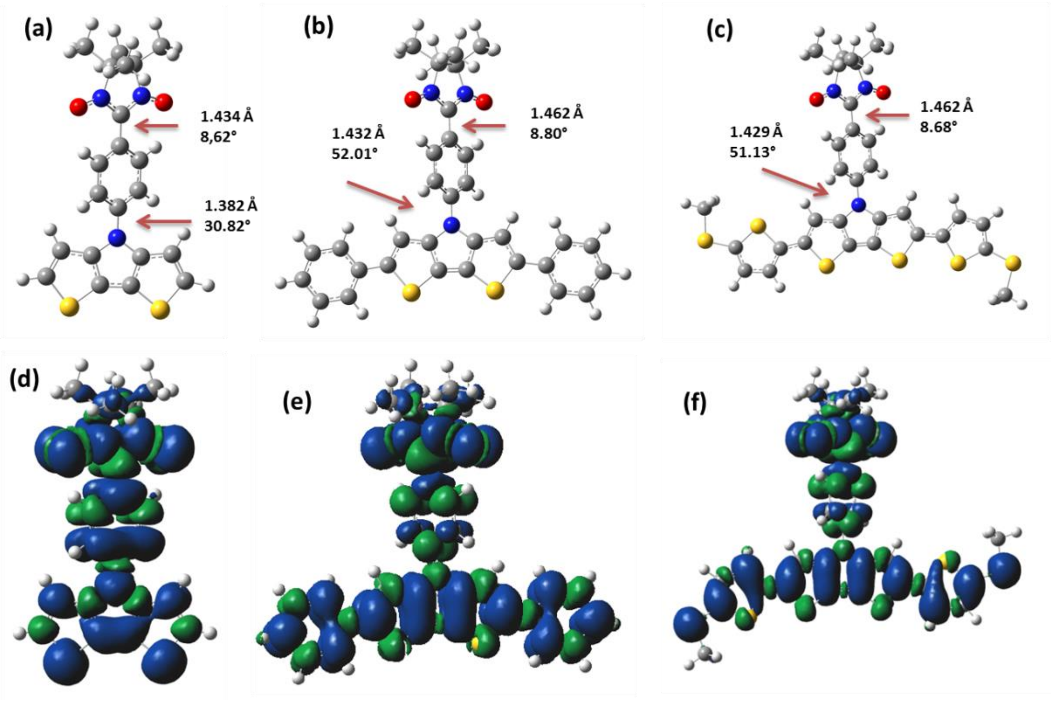

Figure S12: Optimized structures (a), (b), and (c) and spin density distributions (d), (e) and (f) for the **(DTP-Ph-NN)**$^{+\cdot}$, **(Ph_2DTP-Ph-NN)**$^{+\cdot}$, and **(Th_2DTP-Ph-NN)**$^{\cdot+}$respectively. These were calculated by DFT using broken symmetry (BS) approach calculations.

The calculated exchange interactions (J_{intra}/kB) are +5000 K, and +965.4 K for (**Ph_2DTP-Ph-NN**)$^{\cdot+}$ and (**Th_2DTP-Ph-NN**)$^{\cdot+}$ whereas –3580 K for (**DTP-Ph-NN**)$^{\cdot+}$ respectively. The calculated exchange interactions (J_{intra}/kB) are also given in Table S1. For the all the

molecules, the singlet – triplet energy gap is much higher and the energy level of the triplet is lower than the singlet state for **(Ph$_2$DTP-Ph-NN)**$^{\cdot+}$ and **(Th$_2$DTP-Ph-NN)**$^{\cdot+}$ which mean that these molecules are predicted as high spin with triplet species while singlet grout state for **(DTP-Ph-NN)**$^{\cdot+}$. The J_{intra}/kB values are also positive for (**Ph$_2$DTP-Ph-NN**).+ and (**Th$_2$DTP-Ph-NN**)$^{\cdot}$+ , it means that interaction between two spins is ferromagnetic, although for **(DTP-Ph-NN)**$^{\cdot+}$ negative, which means that the magnetic interaction is antiferromagnetic between the NN and radical cation.

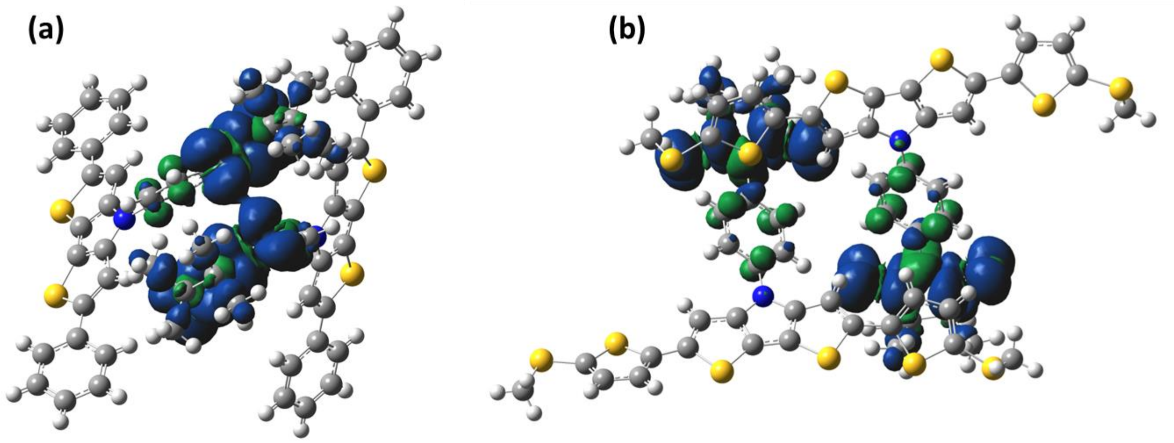

Figure S13: (a) dimer structure of the **(Ph$_2$DTP–PhNN)$_2$** and (b) **(Th$_2$DTP–PhNN)$_2$** for the inter molecular interaction calculation with spin distribution of the molecules.

Table S3: The inter–molecular interaction DFT calculation details.

	HF (triplet)	HF (singlet)	J_{inter}/k_B
**(Ph$_2$DTP–PhNN)$_2$**	– 4768.4743803	–4768.4743794	+ 0.28K
**(Th$_2$DTP–PhNN)$_2$**	–7801.4748581	–7801.474857	–0.35 K

The calculation was carried out by using UBLYP/6-31g(d) basic set in DFT

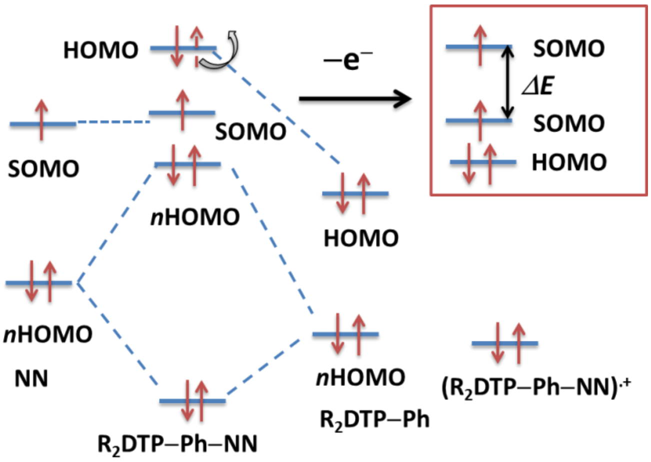

Figure S14: Proposed one electron oxidation mechanism for **R$_2$DTP–Ph–NN.**

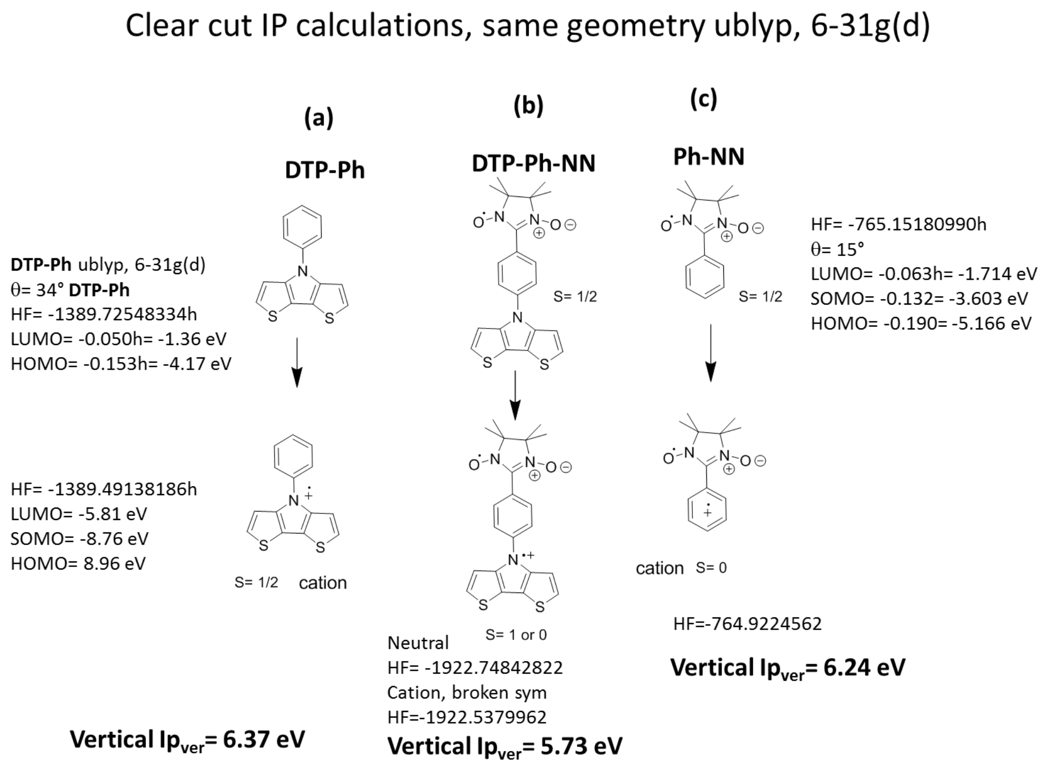

Figure S15: Details of the vertical ionization potential calculation for (a) **DTP-Ph** (b) **DTP-Ph-NN** and (C) **Ph-NN**

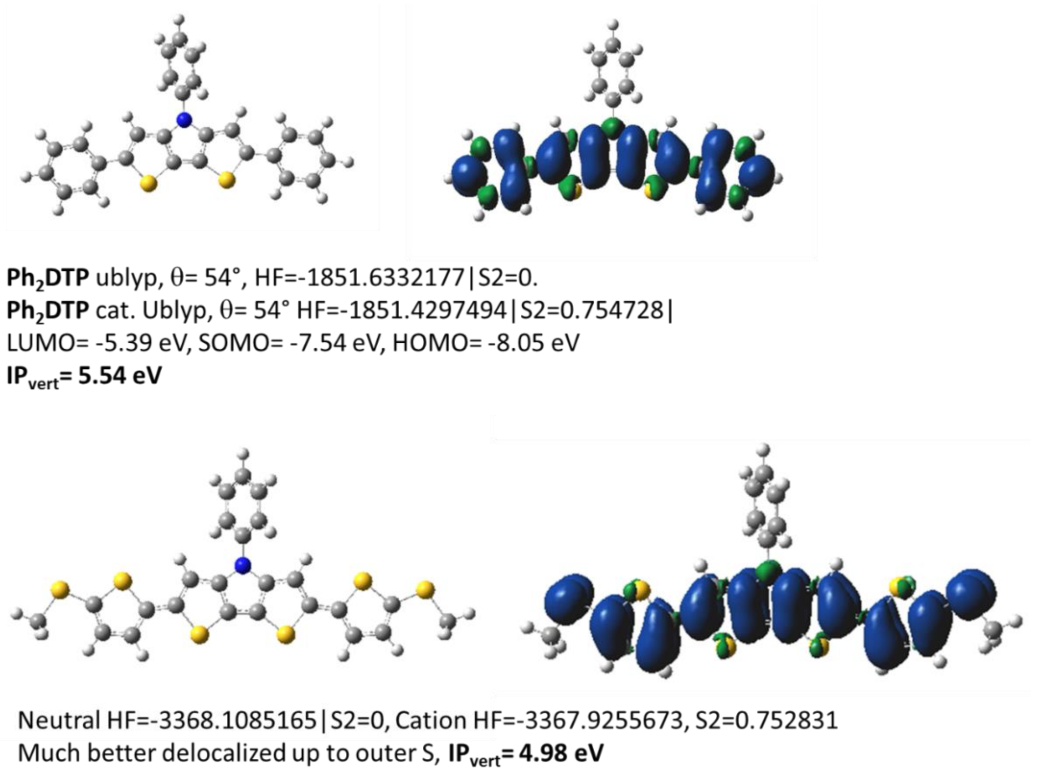

Figure S16: Details of the vertical ionization potential calculation **Ph$_2$DTP-Ph** and (b) **Th$_2$DTP-Ph-NN**

Crystal data for Ph_2DTP-Ph-NN

formula	$C_{33}H_{28}N_3O_2S_2$, C_7H_5N
molecular weight	665.82 $gmol^{-1}$
absorption	μ = 0.20 mm^{-1}
crystal size	0.04 x 0.24 x 0.35 mm^3 blow plate
space group	P bcn (orthorhombic)
lattice parameters	a =24.6446(11)Å
(calculate from	b =8.8218(3)Å
11281 reflections with	c =31.0019(14)Å
1.8° < θ < 28.5°)	V =6740.1(5)$Å^3$ z = 8 F(000) = 2792.0
temperature	-80°C
density	d_{xray} = 1.312 gcm^{-3}

data collection

diffractometer	STOE IPDS 2T
radiation	Mo-K_α Graphitmonochromator
Scan – type	ω scans
Scan – width	1°
scan range	2° ≤ θ < 28°
	-32 ≤ h ≤ 28 -10 ≤ k ≤ 11 -41 ≤ l ≤ 34
number of reflections:	
measured	23518
unique	8175 (R_{int} = 0.0845)
observed	3215 (|F|/σ(F) > 4.0)

data correction, structure solution and refinement

corrections	Lorentz and polarisation correction.
Structure solution	Program: SHELXT-2014
refinement	Program: SHELXL-2017 (full matrix). 438 refined parameters, weighting scheme: $w=1/[\sigma^2(F_o^2) + (0.1313*P)^2]$ with $(Max(F_o^2,0)+2*F_c^2)/3$. H-atoms at calculated positions and refined with isotropic displacement parameters, non H- atoms refined anisotropically.
R-values	wR2 = 0.2598 (R1 = 0.0708 for observed reflections, 0.1819 for all reflections)
goodness of fit	S = 0.912
maximum deviation of parameters	0.001 * e.s.d
maximum peak height in diff. Fourier synthesis	0.33, -0.37 $eÅ^{-3}$

Crystal data for **MeSTh$_2$DTP-Ph-NN**

formula	$C_{30}H_{30}N_3O_2S_6$
molecular weight	656.97 gmol^{-1}
absorption	μ = 0.481 mm^{-1}
crystal size	0.08 x 0.13 x 0.18 mm^3 green block
space group	P -1 (triclinic)
lattice parameters	a =13.3558(12)Å α = 74.063(7)°
(calculate from	b =14.4078(13)Å β = 77.977(7)°
11910 reflections with	c =18.2910(18)Å γ = 65.698(7)°
2.2° < θ < 28.4°)	V = 3066.2(5)Å^3 z = 4 F(000) = 1388
temperature	-80°C
density	d_{xray} = 1.445 gcm^{-3}

data collection

diffractometer	STOE IPDS 2T
radiation	Mo-K$_\alpha$ Graphitmonochromator
Scan – type	ω scans
Scan – width	1°
scan range	2° ≤ θ < 28°
	-17 ≤ h ≤ 17 -18 ≤ k ≤ 19 -24 ≤ l ≤ 24
number of reflections:	
measured	31706
unique	15119 (R_{int} = 0.2423)
observed	5244 (|F|/σ(F) > 4.0)

data correction, structure solution and refinement

corrections	Lorentz and polarisation correction.
Structure solution	Program: SIR-2004 (Direct methods)
refinement	Program: SHELXL-2014 (full matrix). 769 refined parameters, weighting scheme: $w=1/[\sigma^2(F_o^2) + (0.0383*P)^2+24.24*P]$ with $(Max(F_o^2,0)+2*F_c^2)/3$. H-atoms at calculated positions and refined with isotropic displacement parameters, non H- atoms refined anisotropically.
R-values	wR2 = 0.3707 (R1 = 0.1625 for observed reflections, 0.3387 for all reflections)
goodness of fit	S = 1.115
maximum deviation of parameters	0.001 * e.s.d
maximum peak height in diff. Fourier synthesis	0.44, -0.42 eÅ^{-3}
remark	structure contains two independend molecules with different orientation of the thiophene ring

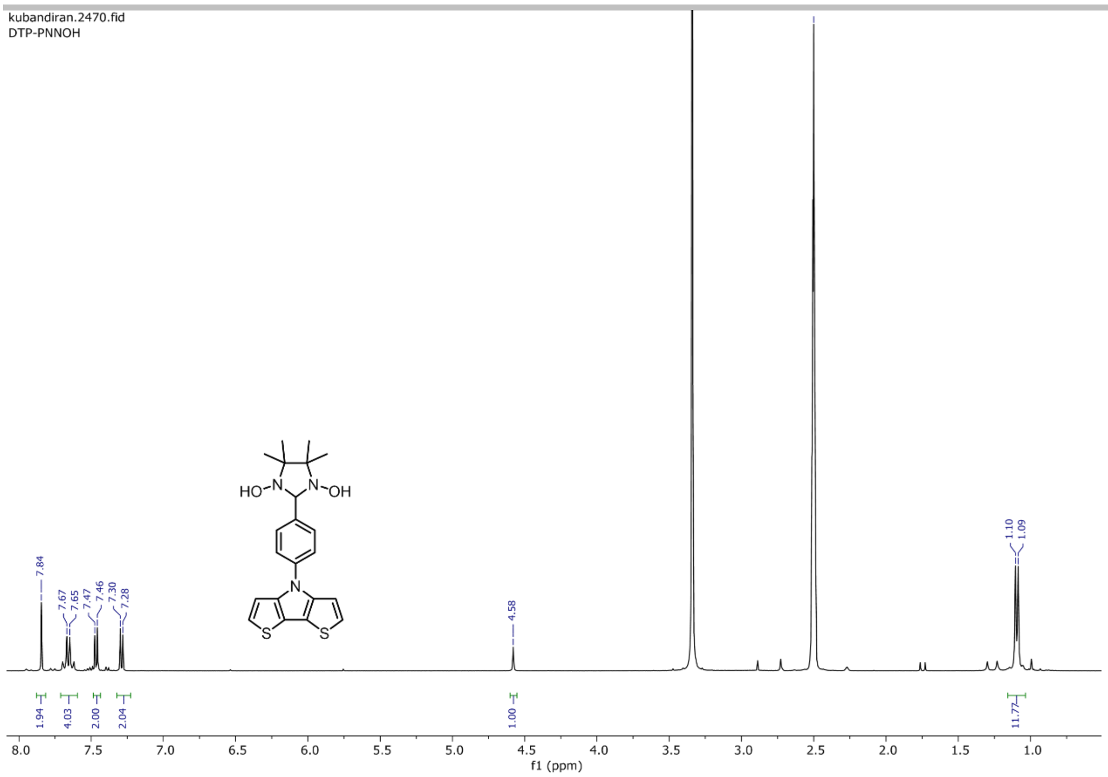

Figure S17: ^{1}H NMR spectrum of **4**

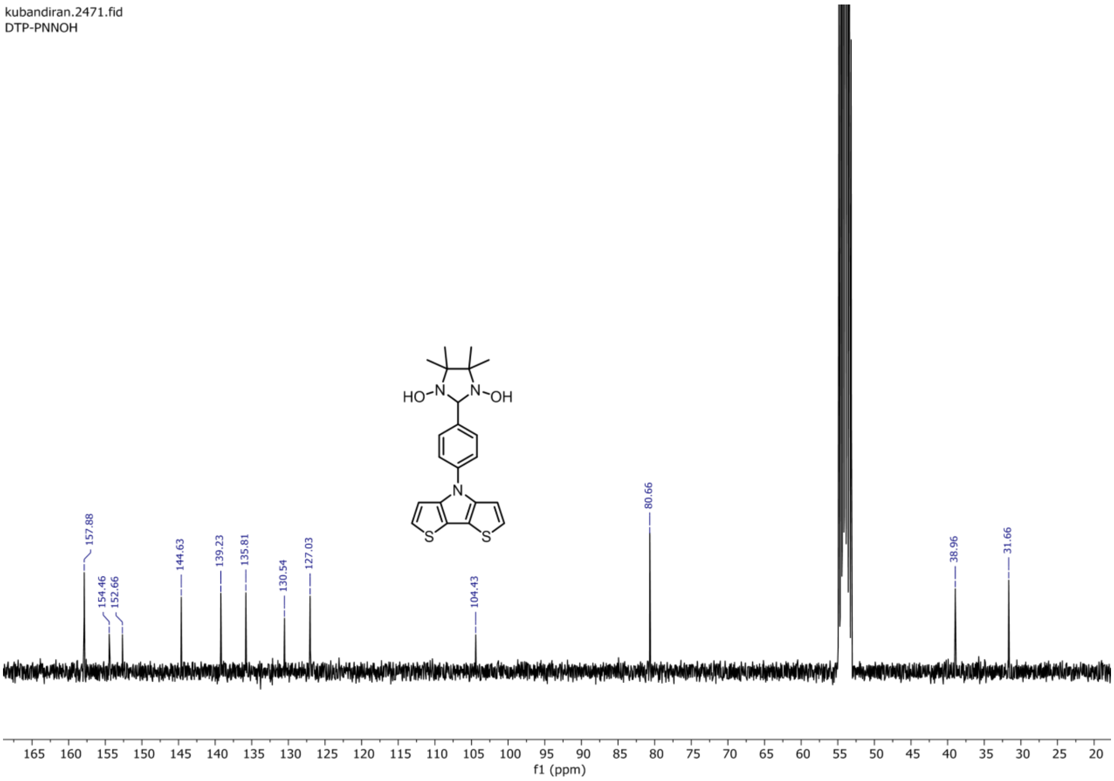

Figure S18: ^{13}C NMR spectrum of **4**

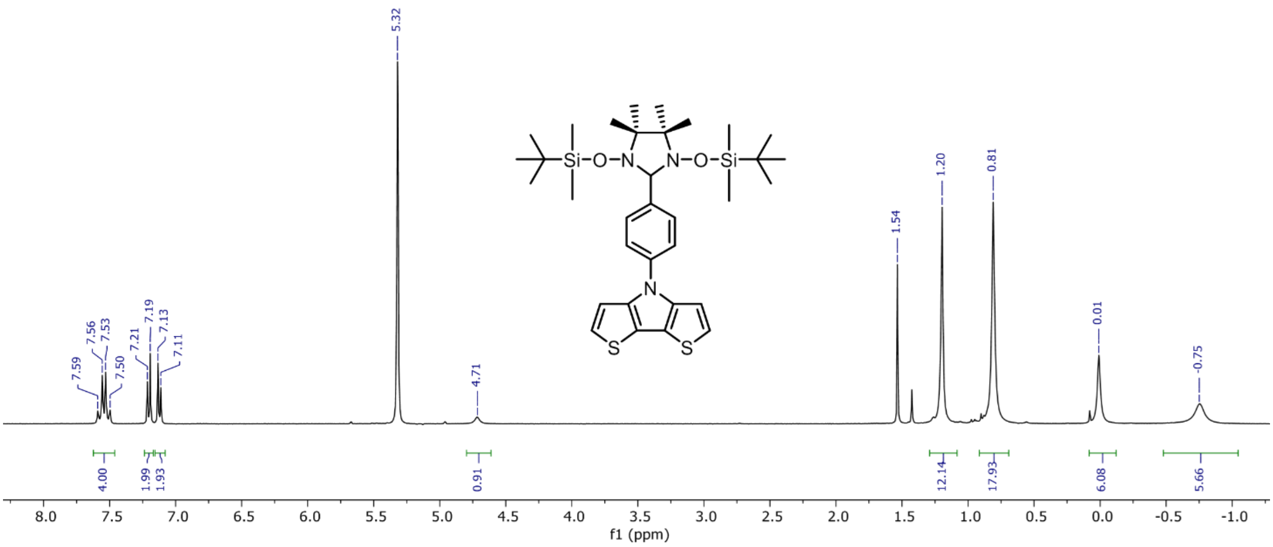

Figure S19: ^{1}H NMR spectrum of **5**

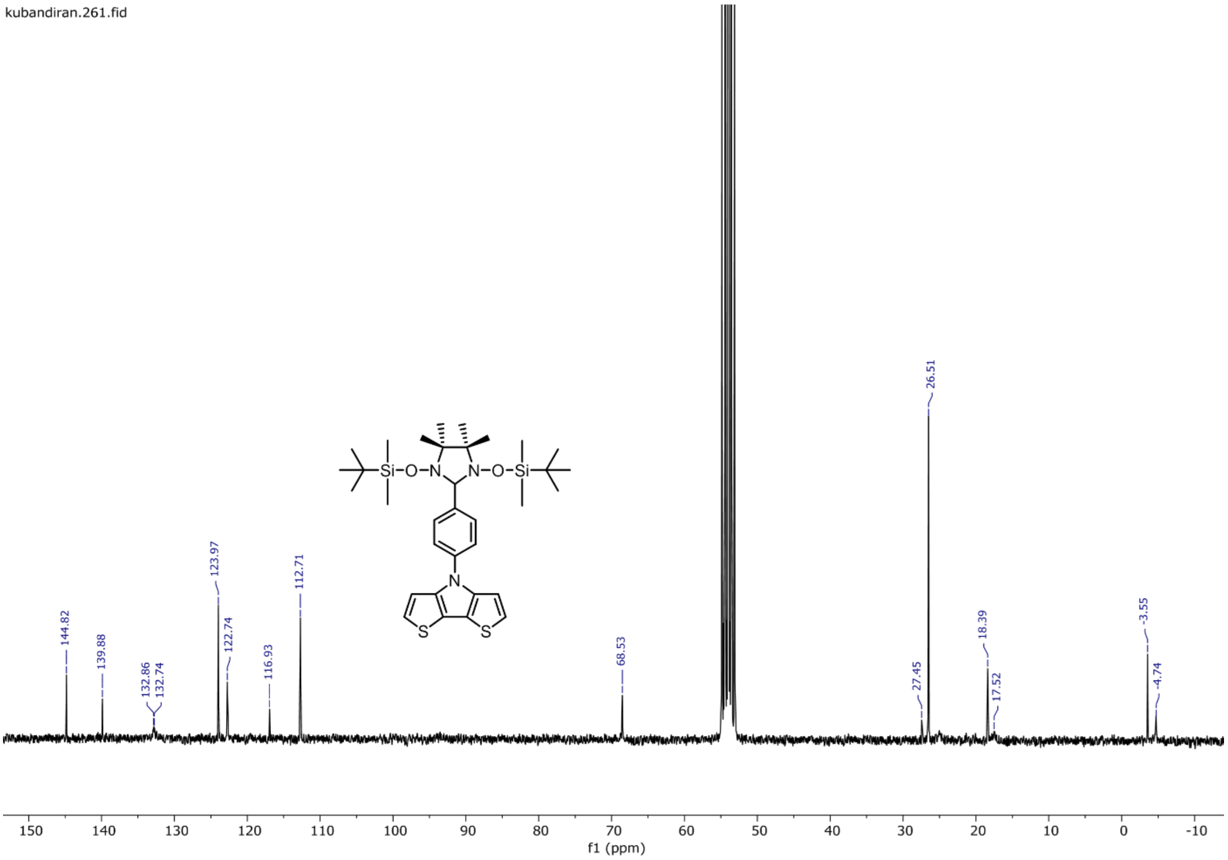

Figure S20: ^{13}C NMR spectrum of **5**

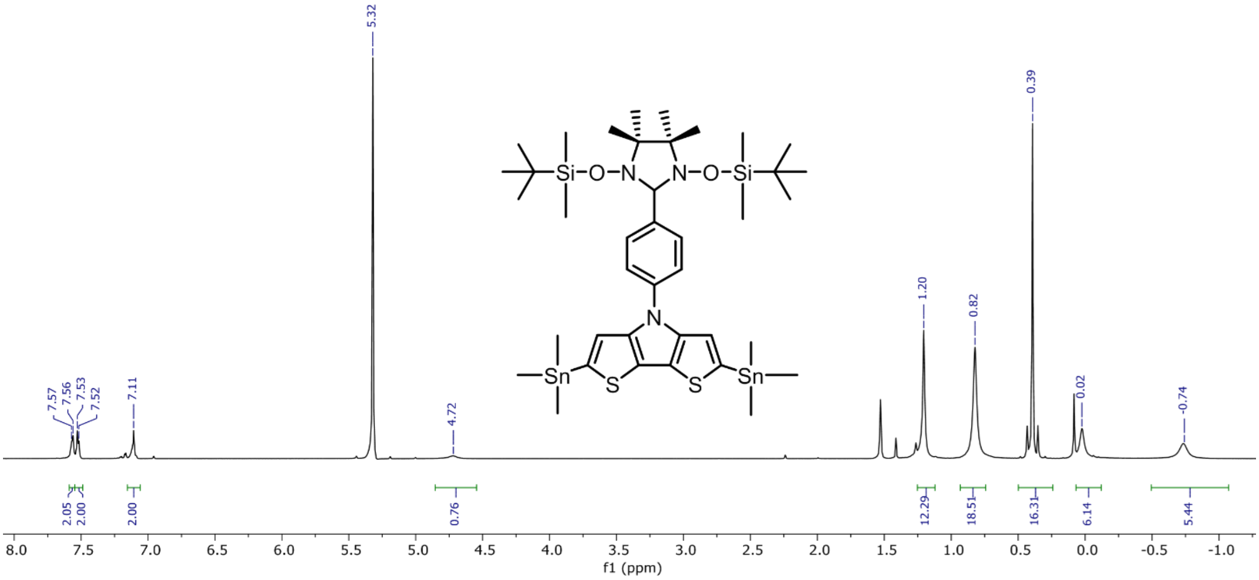

Figure S21: ^{1}H NMR spectrum of **5**

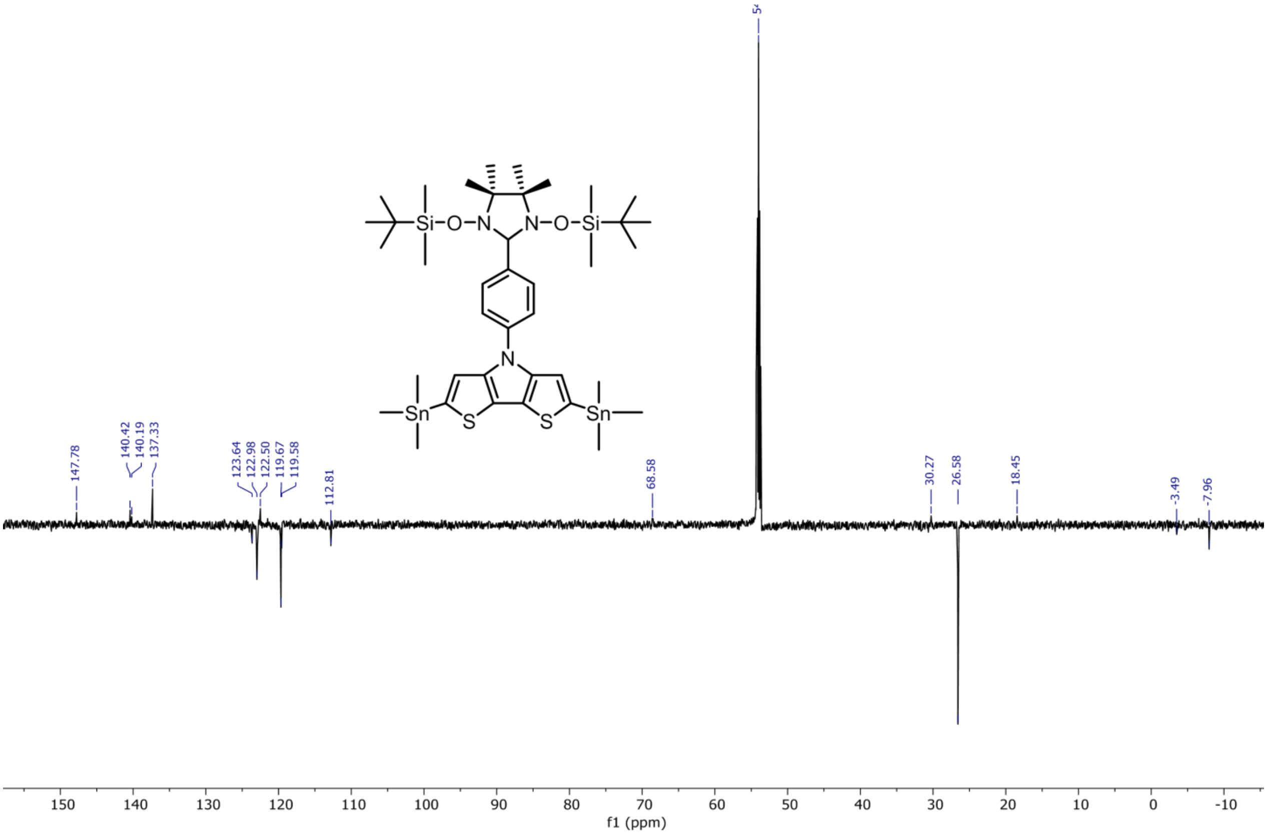

Figure S22: ^{13}C NMR spectrum of **5**

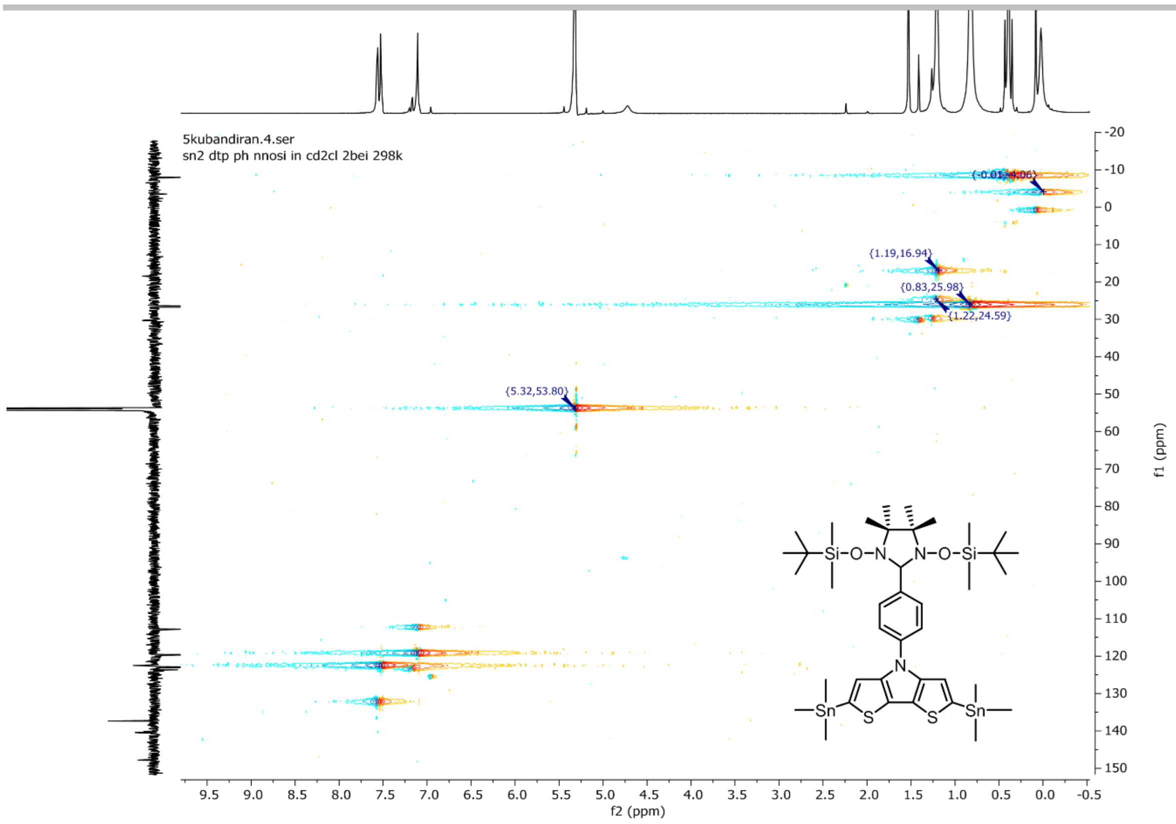

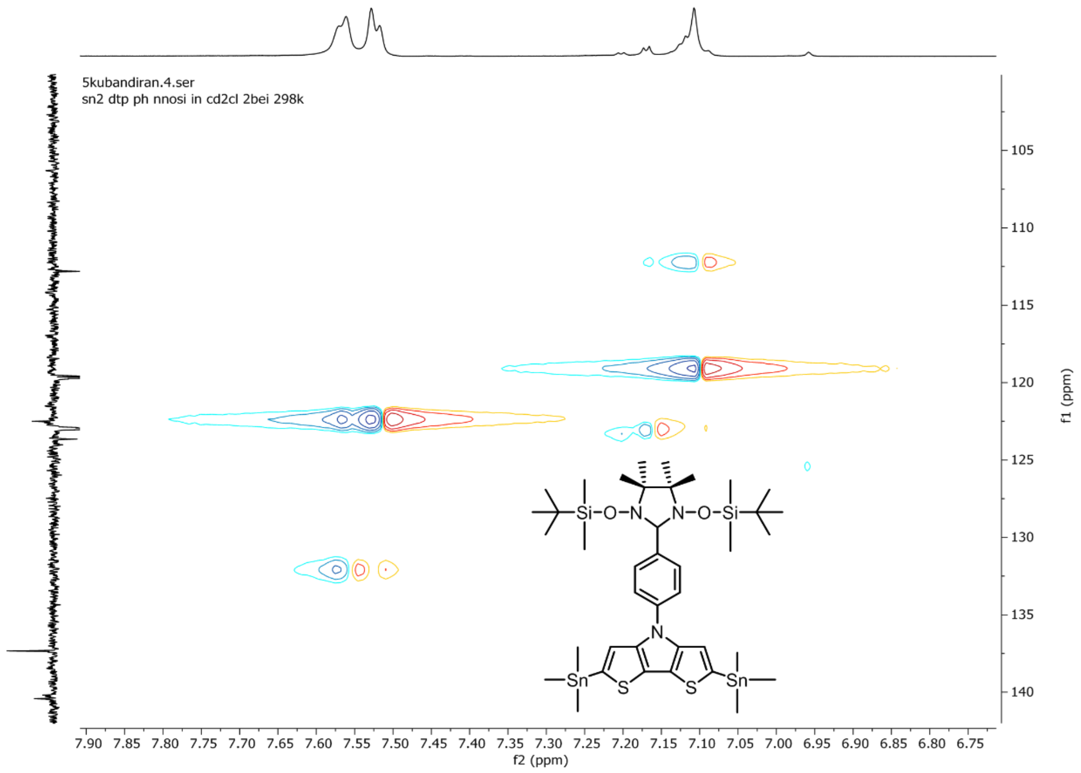

Figure S23: HSQC, 2D-NMR spectrum of **5**

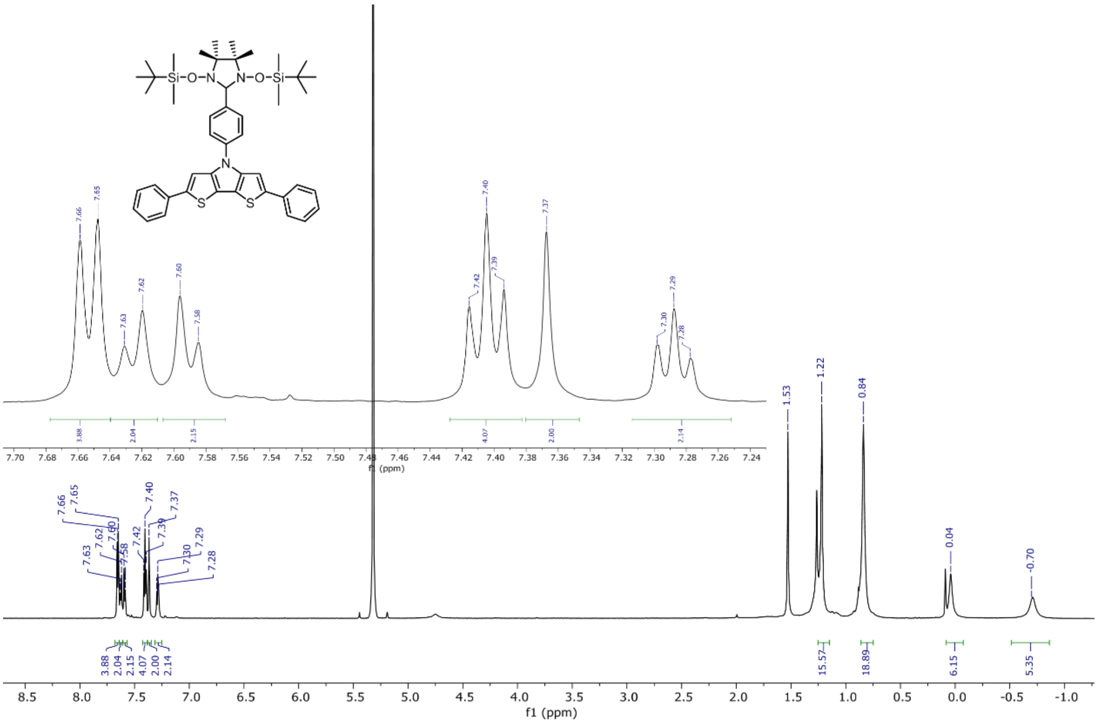

Figure S24: ^{1}H NMR spectrum of **7a**

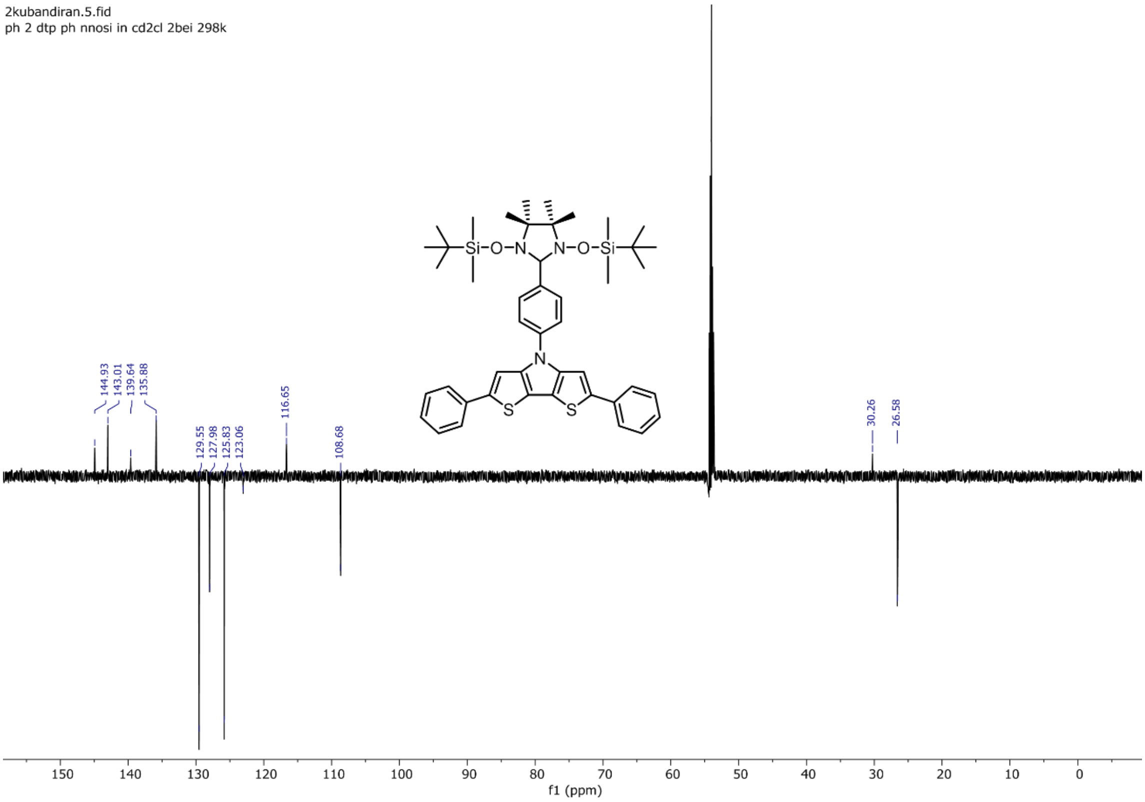

Figure S25: ^{13}C NMR spectrum of **7a**

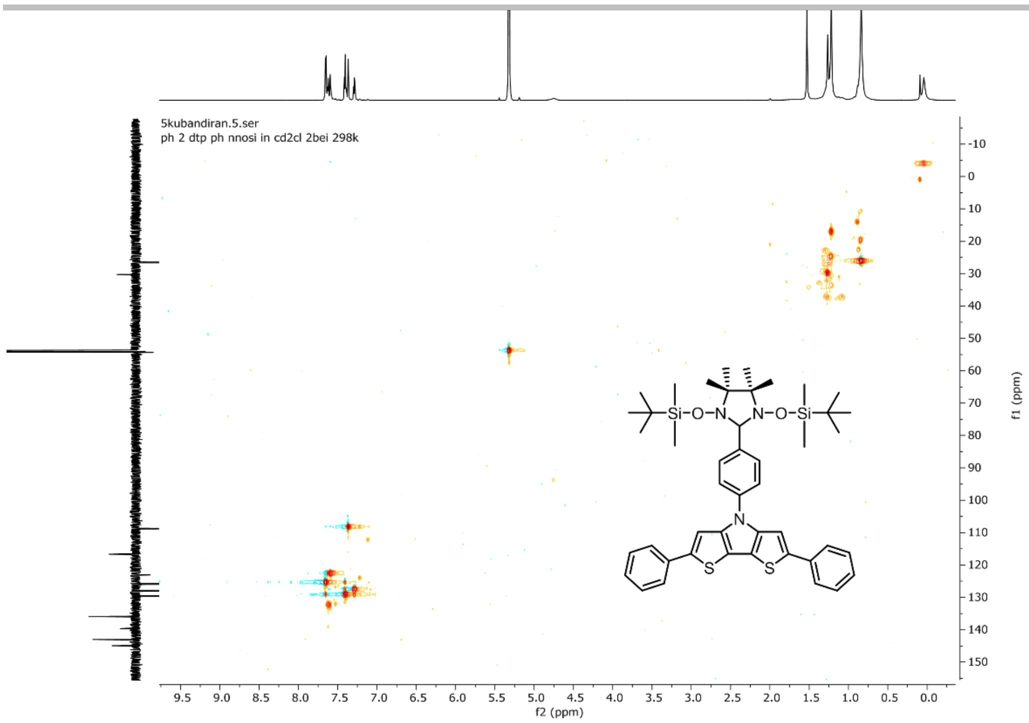

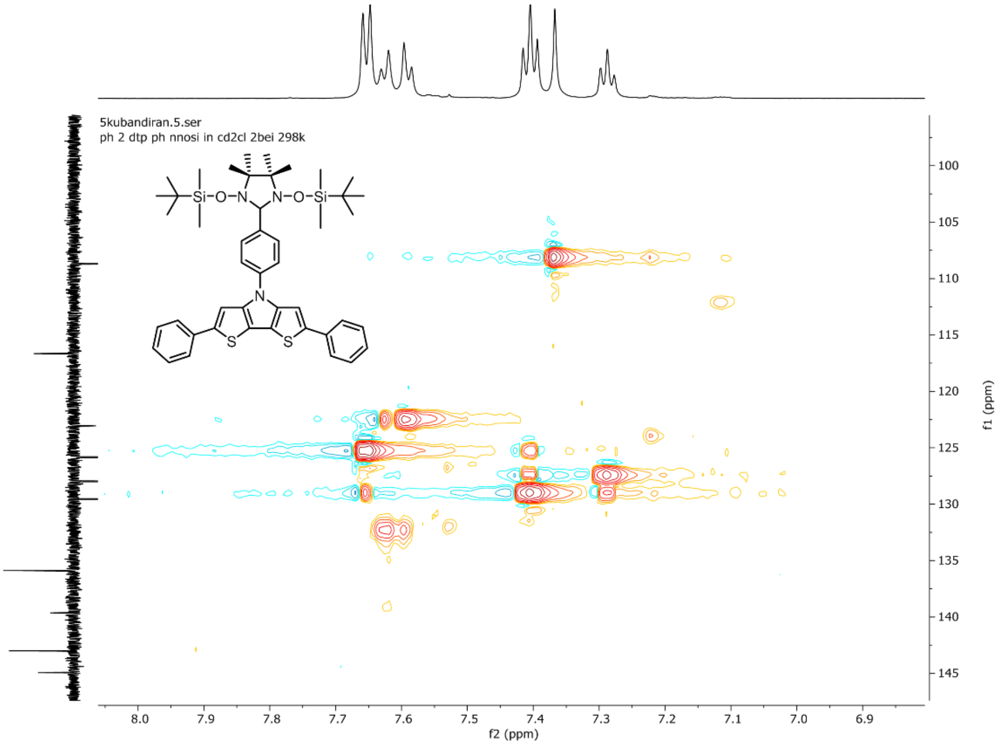

Figure S26: HSQC, 2D-NMR spectrum of **7a**

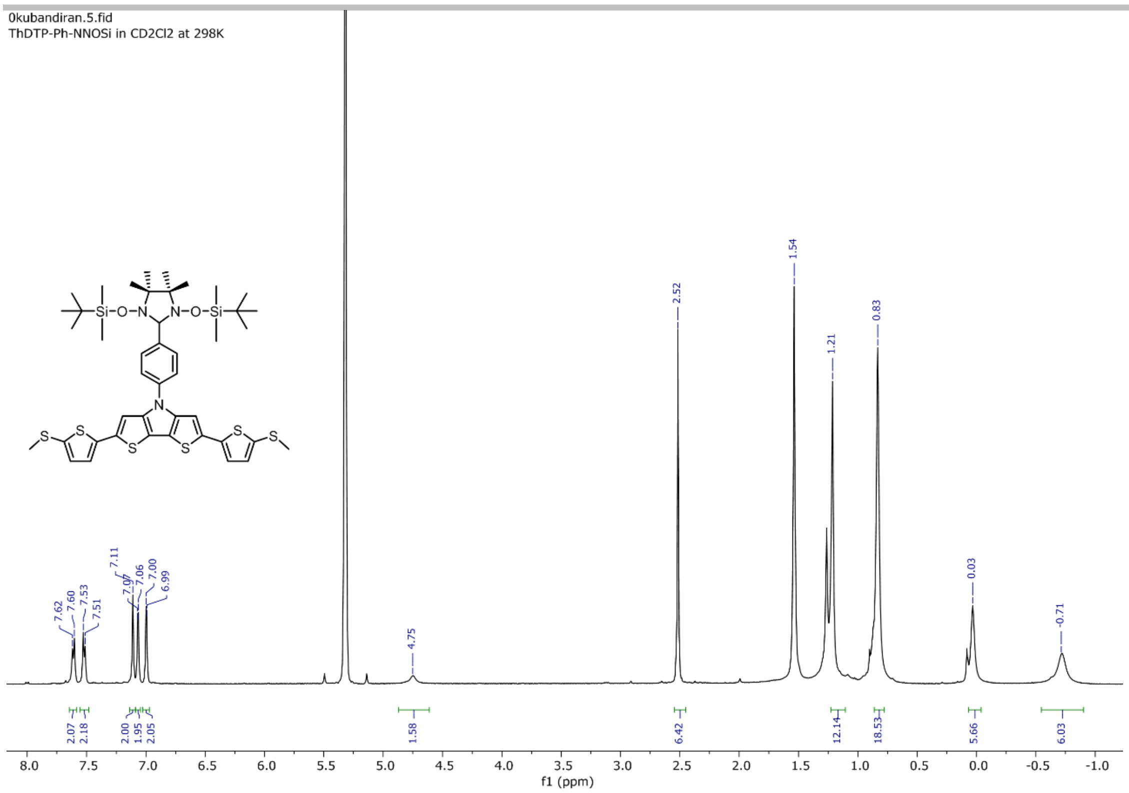

Figure S27: ^{1}H NMR spectrum of **7b**

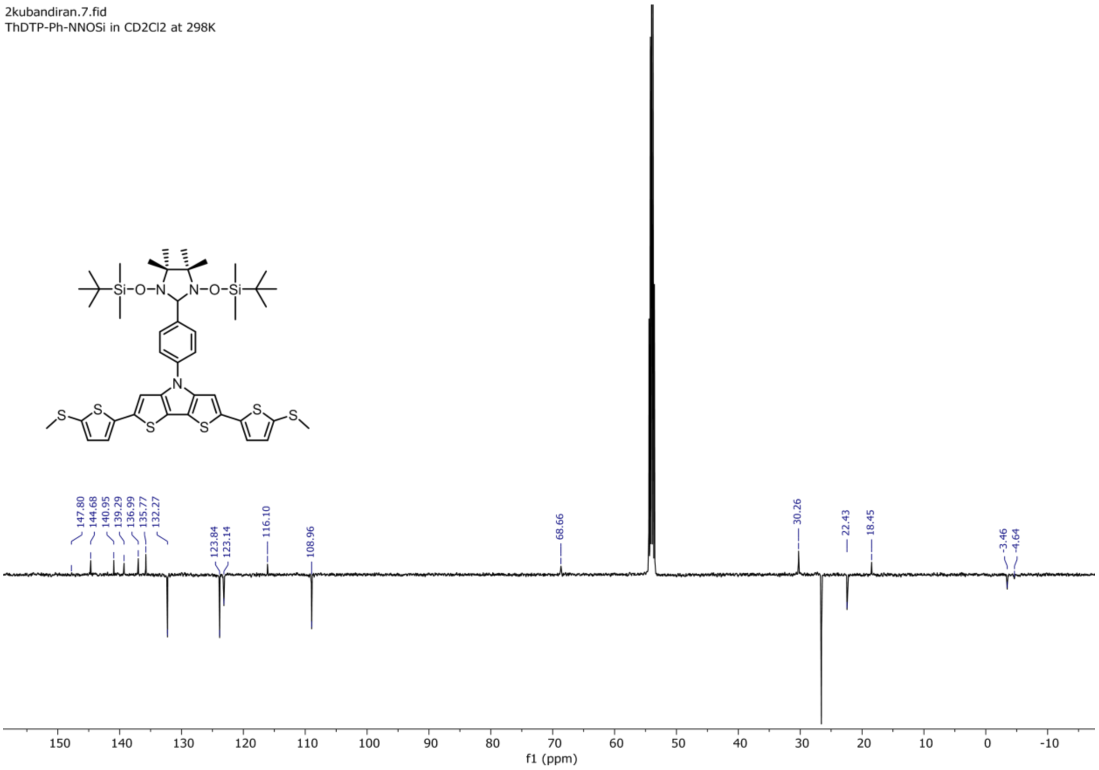

Figure S28: ^{13}C NMR spectrum of **7b**

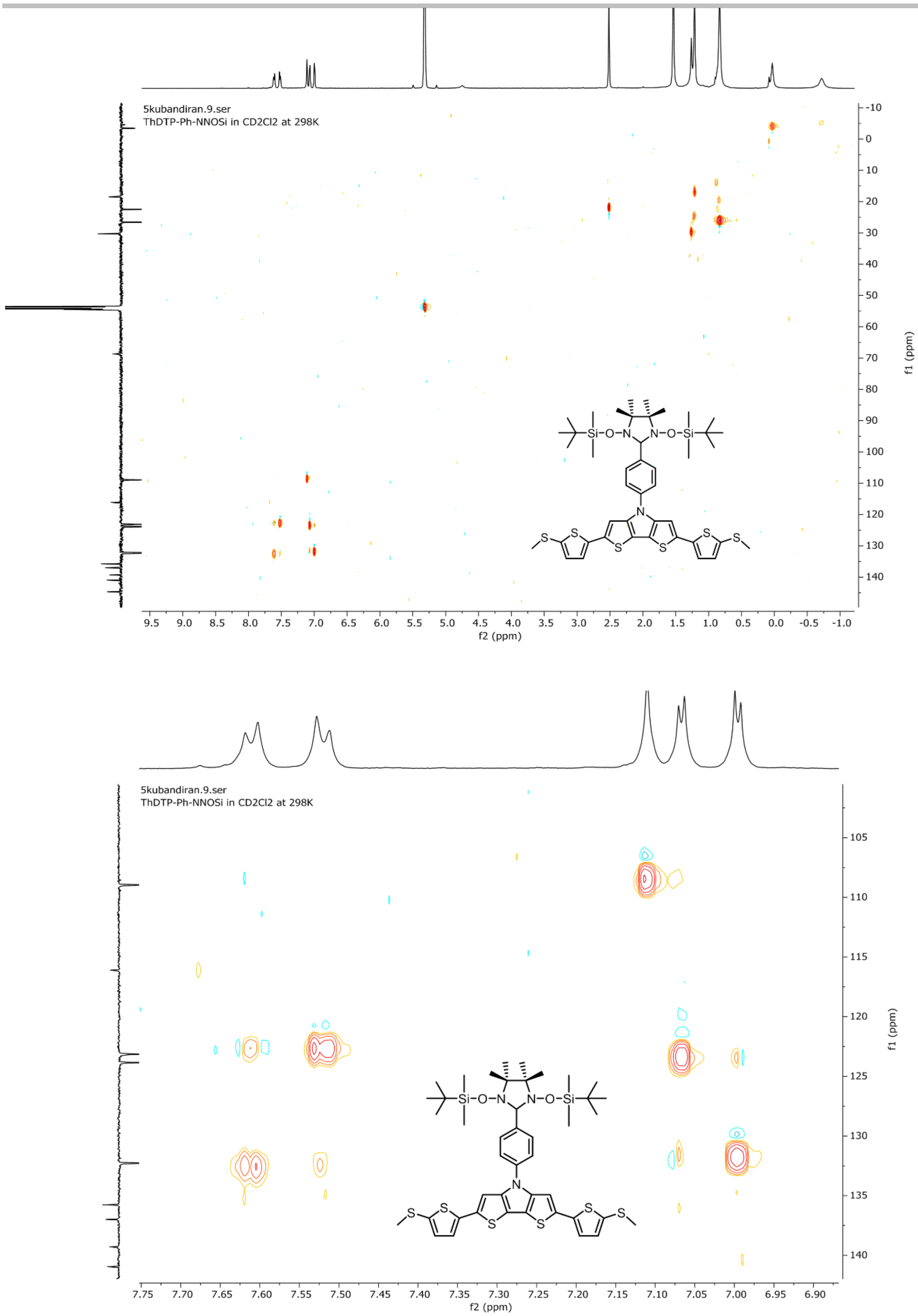

Figure S29: HSQC, 2D-NMR spectrum of **7b**

References S1

[1] J. R. Briggs, J. Klosin, G. T. Whiteker, *O. Lett.* **2005**, *7*, 4795-4798.
[2] J. A. Riddle, X. Jiang, J. Huffman, D. Lee, *Angewandte Chemie International Edition* **2007**, *46*, 7019-7022.
[3] T. Harschneck, N. Zhou, E. F. Manley, S. J. Lou, X. Yu, M. R. Butler, A. Timalsina, R. Turrisi, M. A. Ratner, L. X. Chen, R. P. H. Chang, A. Facchetti, T. J. Marks, *Chemical Communications* **2014**, *50*, 4099-4101.
[4] W. Vanormelingen, L. Pandey, M. Van der Auweraer, T. Verbiest, G. Koeckelberghs, *Macromolecules* **2010**, *43*, 2157-2168.
[5] G. Szalóki, O. Alévêque, J.-L. Pozzo, R. Hadji, E. Levillain, L. Sanguinet, *J. Phys. Chem. B* **2015**, *119*, 307-315.
[6] M. J. Frisch, G. W. Trucks, H. B. Schlegel, G. E. Scuseria, M. A. Robb, J. R. Cheeseman, G. Scalmani, V. Barone, B. Mennucci, G. A. Petersson, H. Nakatsuji, M. Caricato, X. Li, H. P. Hratchian, A. F. Izmaylov, J. Bloino, G. Zheng, J. L. Sonnenberg, M. Hada, M. Ehara, K. Toyota, R. Fukuda, J. Hasegawa, M. Ishida, T. Nakajima, Y. Honda, O. Kitao, H. Nakai, T. Vreven, J. Montgomery, J. A.; Peralta, J. E.; Ogliaro, F.; Bearpark, M.; Heyd, J. J.; Brothers, E.; Kudin, K. N.; Staroverov, V. N.; Kobayashi, R.; Normand, J.; Raghavachari, K.; Rendell, A.; Burant, J. C.; Iyengar, S. S.; Tomasi, J.; Cossi, M.; Rega, N.; Millam, N. J.; Klene, M.; Knox, J. E.; Cross, J. B.; Bakken, V.; Adamo, C.; Jaramillo, J.; Gomperts, R.; Stratmann, R. E.; Yazyev, O.; Austin, A. J.; Cammi, R.; Pomelli, C.; Ochterski, J. W.; Martin, R. L.; Morokuma, K.; Zakrzewski, V. G.; Voth, G. A.; Salvador, P.; Dannenberg, J. J.; Dapprich, S.; Daniels, A. D.; Farkas, Ö.; Foresman, J. B.; Ortiz, J. V.; Cioslowski, J.; Fox, D. J., , (Ed.: Gaussian Inc.: Wallingford CT), **2009**.
[7] aK. Kolanji, P. Ravat, A. S. Bogomyakov, V. I. Ovcharenko, D. Schollmeyer, M. Baumgarten, *J. Org. Chem* **2017**, *82*, 7764-7773; bP. Ravat, Y. Borozdina, Y. Ito, V. Enkelmann, M. Baumgarten, *Crystal Growth & Design* **2014**, *14*, 5840-5846; cY. B. Borozdina, E. Mostovich, V. Enkelmann, B. Wolf, P. T. Cong, U. Tutsch, M. Lang, M. Baumgarten, *J. Mater. Chem. C* **2014**, 6618-6629.
[8] aL. Noodleman, *J. Chem. Phys.* **1981**, *74*, 5737-5743; bL. Noodleman, E. R. Davidson, *Chem. Phys.* **1986**, *109*, 131-143; cK. Yamaguchi, F. Jensen, A. Dorigo, K. N. Houk, *Chem. Phys. Lett.* **1988**, *149*, 537-542; dT. Soda, Y. Kitagawa, T. Onishi, Y. Takano, Y. Shigeta, H. Nagao, Y. Yoshioka, K. Yamaguchi, *Chem. Phys. Lett.* **2000**, *319*, 223-230; eM. Shoji, K. Koizumi, Y. Kitagawa, T. Kawakami, S. Yamanaka, M. Okumura, K. Yamaguchi, *Chem. Phys. Lett.* **2006**, *432*, 343-347.
